# Nuclear speckle specific hnRNP D-like prevents age- and AD-related cognitive decline by modulating RNA splicing

**DOI:** 10.1186/s13024-021-00485-w

**Published:** 2021-09-22

**Authors:** Qingyang Zhang, Juan Zhang, Jin Ye, Xiaohui Li, Hongda Liu, Xiaolin Ma, Chao Wang, Keqiang He, Wei Zhang, Ji Yuan, Yingjun Zhao, Huaxi Xu, Qiang Liu

**Affiliations:** 1 Institute on Aging and Brain Disorders, The First Affiliated Hospital of USTC, Hefei National Laboratory for Physical Sciences at the Microscale, Division of Life Sciences and Medicine, University of Science and Technology of China, Hefei, 230026 China; 2 Anhui Province Key Laboratory of Biomedical Aging Research, University of Science and Technology of China, Hefei, 230026 China; 3 School of Life Sciences, Division of Life Sciences and Medicine, University of Science and Technology of China, Hefei, 230026 China; 4 Department of Anesthesiology, The First Affiliated Hospital of USTC, Division of Life Sciences and Medicine, University of Science and Technology of China, Hefei, 230001 China; 5 The First Affiliated Hospital of Xiamen University, Fujian Provincial Key Laboratory of Neurodegenerative Disease and Aging Research, Institute of Neuroscience, Xiamen University, Xiamen, 361000 China; 6 Center for Excellence in Animal Evolution and Genetics, Chinese Academy of Sciences, Kunming, 650201 China

**Keywords:** hnRNP DL, Aging, Alzheimer’s disease (AD), Cognition, Nuclear speckles, Alternative splicing

## Abstract

**Background:**

Aberrant alternative splicing plays critical role in aging and age-related diseases. Heterogeneous nuclear ribonucleoproteins (hnRNPs) reportedly regulate RNA splicing process. Whether and how hnRNPs contribute to age-related neurodegenerative diseases, especially Alzheimer’s disease (AD), remain elusive.

**Methods:**

Immunoblotting and immunostaining were performed to determine expression patterns and cellular/subcellular localization of the long isoform of hnRNP D-like (L-DL), which is a hnRNP family member, in mouse hippocampus. Downregulation of L-DL in WT mice was achieved by AAV-mediated shRNA delivery, followed by memory-related behavioural tests. L-DL interactome was analysed by affinity-precipitation and mass spectrometry. Alternative RNA splicing was measured by RNA-seq and analyzed by bioinformatics-based approaches. Downregulation and upregulation of L-DL in APP/PS1 mice were performed using AAV-mediated transduction.

**Results:**

We show that L-DL is specifically localized to nuclear speckles. L-DL levels are decreased in the hippocampus of aged mouse brains and downregulation of L-DL impairs cognition in mice. L-DL serves as a structural component to recruit other speckle proteins, and regulates cytoskeleton- and synapse-related gene expression by altering RNA splicing. Mechanistically, these splicing changes are modulated via L-DL-mediated interaction of SF3B3, a core component of U2 snRNP, and U2AF65, a U2 spliceosome protein that guides U2 snRNP’s binding to RNA. In addition, L-DL levels are decreased in APP/PS1 mouse brains. While downregulation of L-DL deteriorates memory deficits and overexpression of L-DL improves cognitive function in AD mice, by regulating the alternative splicing and expression of synaptic gene *CAMKV*.

**Conclusions:**

Our findings define a molecular mechanism by which hnRNP L-DL regulates alternative RNA splicing, and establish a direct role for L-DL in AD-related synaptic dysfunction and memory decline.

**Supplementary Information:**

The online version contains supplementary material available at 10.1186/s13024-021-00485-w.

## Background

Alternative splicing is a process wherein specific regulators modulate core splicing machinery to selectively generate various splicing variants [1]. Pre-mRNA splicing relies on recruitment of a large ribonucleoprotein complex, known as spliceosome, at 5′ and 3′ splice sites (5′SS and 3′SS). Typically, the 3′SS is bound by U2 auxiliary factors (U2AFs) in spliceosome assembly, wherein U2AF small subunit U2AF35 binds to the 3′ SS, and the large subunit U2AF65 binds to the polypyrimidine tract adjacent to the 3′ SS [2]. U2AF65 interacts with core U2 small nuclear ribonucleoprotein (U2 snRNP) component splicing factor 3B (SF3B), such as SF3B1, SF3B2 or SF3B3, to promote binding of U2 snRNP to branch sites and subsequent splicing complex formation and activation, eventually intron removal [3].

Brain aging is an irreversible physiological process, accompanied by decreased cognitive function and memory loss [4]. Aging is a major risk factor for the progression of neurodegenerative diseases [5, 6]. Mounting evidence has shown a global change in alternative splicing during brain aging and age-related neurodegenerative diseases [7]. Moreover, aberrant splicing produces irregular splicing products with defective or even harmful functions, and contributes to aging or age-related diseases [8]. For instance, exon 18 skipping of postsynaptic density protein 95 (PSD95) results in generation of a splicing variant with premature translational termination, and this splicing variant is prone to degradation through nonsense-mediated decay (NMD) pathway, resulting in reduced levels of PSD95 [9]. This splicing change of PSD95 is promoted by polypyrimidine tract binding protein 1 (PTBP1), whose levels are significantly increased upon aging progression [10]. These findings suggest that age-dependent RNA binding protein-mediated alternative splicing is responsible for alterations in synaptic structure and function during aging progression. *BACE1*, which encodes β-secretase 1 that catalyzes amyloid precursor protein into β-amyloid (Aβ), undergoes extensive alternative splicing upon aging [11, 12]. As a result, full-length BACE1 shows an age-dependent increase, resulting in a rise in BACE1 level and a consequent accumulation of Aβ in the brain [13]. This leads to age-related cognitive impairment and AD progression [14–16]. Notably, a mutation in intron 4 of presenilin 1 (PS1) leads to production of an aberrant transcript and a full-length PS1 with insertion of an extra threonine, consequently promoting Aβ42 production and AD pathology [17, 18].

Nuclear speckles, also known as SC35 domain, are originally discovered as a storage site for RNA processing factors with functions predominantly linked to the alternative splicing of pre-mRNA [19]. The shape and size of nuclear speckles are in a dynamic change due to continual exchange of splicing factors between speckles and nucleoplasm [20]. Cellular processes such as Pol II transcription and splicing are involved in dynamic changes of nuclear speckles [21]. Heterogeneous nuclear ribonucleoproteins (hnRNPs), a classic splicing regulator, are extensively involved in alternative splicing regulation [1]. Biochemical purification and proteomic analyses have identified the existence of multiple hnRNPs in nuclear speckles, which may contribute to maintaining the structural and functional integrity of nuclear speckles [22, 23].

Here, we demonstrate that L-DL is predominantly expressed in neurons and its levels are significantly decreased in the hippocampus of both aged and AD brains. Particularly, deficiency of L-DL in the hippocampus impairs cognitive function. We uncover that L-DL is specifically localized to nuclear speckles, serving as a structural component to maintain the structure of this nuclear body. Moreover, L-DL regulates splicing patterns of multiple cytoskeleton- and synapse-related genes via modulating the loading of U2 snRNP-mediated spliceosome on their pre-mRNAs. In addition, L-DL levels are reduced in APP/PS1 (AD model) mouse brains, and further downregulation of L-DL in these mice deteriorates memory decline, whereas overexpression of L-DL restores synaptic protein expressions and cognitive function in AD mice. Taken together, these findings suggest that nuclear speckle specific L-DL regulates aging- and AD-related cognitive function via modulating alternative splicing of cytoskeleton- and synapse-related genes.

## Methods

### Cell culture and plasmid transfection

Neuro-2a cells (N2a) (ATCC, CCL-131) and 293T cells (ATCC, CRL-3216) were cultured according to standard procedures. Cells were growing in DMEM (Gibco, #12100061) supplemented with 10% FBS (Biological Industries, #1827594) and 1% penicillin/streptomycin (Gibco, #15070063) and incubated in 5% humidified CO_2_ incubator at 37 °C. Plasmid transfection was performed when cells were approximately 70–80% confluent using PEI (Sigma-Aldrich, #408727).

### Animals

C57BL/6 J mice were purchased from GemPharmatech and APP/PS1 mice were purchased from Shanghai Model Organisms Center. All experimental protocols were approved by the Animal Studies Committee at University of Science and Technology, Hefei, China.

### Plasmid construction

To generate pcDNA 3.1 (+)-GST vector, GST sequence was PCR amplified from pcDNA3.1 + N-GST (TEV) (GenScript) and subcloned into Nhe I / EcoR I restriction sites of pcDNA 3.1 (+) (Thermo Fisher Scientific, #V790–20). To generate pcDNA 3.1 (+)-GST-L-DL plasmid, the coding sequence of L-DL was PCR amplified from cDNA derived from mouse hippocampus, then subcloned into EcoR I / Xho I restriction sites of pcDNA 3.1 (+)-GST vector. Primers for cloning are listed in Table S1.

Splicing reporter was constructed according to previously published [24]. Luciferase-intron-Mutant (luciferase-Mut), which was generated by inserting an immunoglobulin intron (Promega, #U47119) into firefly luciferase gene, and wild-type firefly luciferase (luciferase-WT) with PEST and CL1 sequence for protein destabilization were synthesized (Tsingke) and subcloned into BamH I / EcoR I restriction sites of pcDNA 3.1 (+) vector.

For adeno-associated virus (AAV) plasmid expressing L-DL shRNA, four H1 promoter driven L-DL shRNA sequences were tandemly arranged and synthesized (Tsingke), then subcloned into BamH I / EcoR I restriction sites of pAAV-EF1a-DIO-Gcamp6s vectors (Addgene, #67526), wherein EF1a promoter was substituted with CamkIIα promoter followed by zsGreen sequence for neuron-specific visualization of the injected areas. AAV was produced and microinjected into the hippocampus of mouse brains for knockdown. The sequences of luciferase-WT, luciferase-Mut and four tandemly arranged L-DL shRNA sequences are listed in Table S2.

L-DL sgRNAs were designed using online sgRNA designing tools: https://sg.idtdna.com/site/order/designtool/index/CRISPR_SEQUENCE. Four mouse sgRNAs and four human sgRNAs were separately subcloned into the BsmB I site of LentiCRISPR v2 plasmid (Addgene, #52961) as previously described [25, 26]. These sgRNA sequences are listed in Table S3.

### CRISPR-Cas9 mediated L-DL knockout in cells

Mixture of four L-DL-sgRNA on lentiCRISPR plasmid were co-transfected into 293T cells, together with packaging plasmid (pHR’8.2deltaR) and envelope plasmid (pCMV-VSV-G). Virus-containing medium was harvested 48 h after the transfection. Cells were treated with virus containing medium for 48 h. Transduced cells were selected with 10 μg/ml of puromycin.

### Protein extraction and Western blot

Cells and brain tissues were lysed in PBS plus 1% Trion X-100 and 1% protease inhibitor cocktail (Targetmol, #C0001), following by sonication. Protein concentration was measured with BCA Protein Assay Kit (Thermo Fisher Scientific, #23250) and equal amount of protein was separated  by SDS-PAGE electrophoresis. Proteins were then transferred from gel to 0.45 μm nitrocellulose membrane (Pall Corporation, #27182369). Membranes were blocked with 5% non-fat milk in Tris buffered saline containing 0.1% Tween 20 (TBST) for 1 h, following by incubation with primary antibodies at 4 °C overnight. The following primary antibodies were used: rabbit anti-L-DL (Sigma-Aldrich, #HPA063147) at dilution of 1:2,000, rabbit anti-hnRNP DL (Abcepta, #AP5352c) at dilution of 1:1,000, mouse anti-GST (Santa Cruz Biotechnology, #sc-138) at dilution of1:5,000, mouse anti-RBM25 (Santa Cruz Biotechnology, #sc-374271) at dilution of 1:500, mouse anti-NPM1 (Proteintech, #60096-1-Ig) at dilution of 1:2,000, mouse anti-PML (Abcam, #ab6263) at dilution of 1:1,000, mouse anti-SF3B1 (Santa Cruz Biotechnology, #sc-514655) at dilution of 1:1,000 dilution, mouse anti-U2AF65 (Santa Cruz Biotechnology, #sc-53942) at dilution of 1:1,000, mouse anti-U2AF35 (Proteintech, #60289-1-Ig) at dilution of 1:2,500, rabbit anti-SF3B3 (Proteintech, #14577-1-AP) at dilution of 1:2,000, rabbit anti-PSD95 (Proteintech, #20665-1-AP) at dilution of1:1,000, rabbit anti-SNAP25 (Proteintech, #14903-–1-AP) at dilution of 1:5,000, rabbit anti-CAMKV (Proteintech, #14788-1-AP) at dilution of 1:1,000, rabbit anti-NR2B (Proteintech, #21920-1-AP) at  dilution of 1:2,000, rabbit anti-SC35 (Abclonal, #A3635) at dilution of 1:1,000000 dilution, mouse anti-Lamin B1 (Proteintech, #66095-1-Ig) at dilution of 1:5,000, mouse anti-GAPDH (Proteintech, #60004-1-Ig) at dilution of 1:5,000 , rabbit anti-Lamin A/C (Proteintech, #10298-1-AP) at dilution of 1:5,000 . Goat anti-mouse IgG (H + L) (peroxidase/HRP conjugated) (Elabscience, #E-AB-1001) or goat anti-rabbit IgG (H + L) (peroxidase/HRP conjugated) (Elabscience, # E-AB-1003) secondary antibodies were used at dilution of 1:5,000. The immunoreactive bands were detected by Pierce ECL Western Blotting Substrate (Thermo Fisher Scientific, #32209). For densitometric analyses, immunoreactive bands were quantified using Fiji software (https://imagej.nih.gov/ij/).

### Immunofluorescence staining

Cells seeded on cover slips were washed 3 times with PBS, following by fixation in 4% PFA for 20 min. Cells were permeabilized with PBS containing 0.4% Triton X-100 (PBST), following by blocking with 2% BSA in PBST for 30 min. After blocking, cells were incubated with rabbit anti-L-DL (Sigma-Aldrich, #HPA063147) at dilution of 1:200, mouse anti-SC35 (BD Bioscience, #556363) at dilution of a 1:200, mouse anti-SON (Santa Cruz Biotechnology, #sc-398508) at dilution of 1:200, mouse anti-RBM25 (Santa Cruz Biotechnology, #sc-374271) at dilution of 1:50, mouse anti-NPM1 (Proteintech, #60096-1-Ig) at dilution of 1:200, mouse anti-PML (Abcam, #ab6263) at dilution of 1:50, mouse anti-SMN (Abcam, #ab5831) at dilution of 1:50 overnight at 4 °C, followed by incubation with Alexa Fluor 594-conjugated Goat anti-rabbit secondary antibody (Thermo Fisher Scientific, #R37117), or Alexa Fluor 488-conjugated Goat anti-mouse secondary antibody (Thermo Fisher Scientific, #R37120), and 4′, 6-diamidino-2-phenylindole dihydrochloride (DAPI) (Sigma-Aldrich, #D9542).

Immunostaining for brain tissues was performed as described previously, with modifications [27]. Mice were anesthetized and sacrificed, then perfused with 0.9% NaCl solution. Brains were isolated, immersed in 4% paraformaldehyde (PFA) and cryo-preserved in 30% sucrose for 24 h at 4 °C. Tissues were then embedded in OCT compound and sectioned with microtome at 10 μm thickness (Leica, CM1860). Glass slide-mounted sections were washed with TBS (20 mM Tris, 150 mM NaCl, pH 7.2), permeabilized with TBS containing 0.25% Triton X-100, followed by blocking with 0.5% BSA in TBST buffer (20 mM Tris, 150 mM NaCl, 0.1% Triton X-100, pH 7.2) for 1 h. Sections were then incubated with rabbit anti-L-DL (Sigma-Aldrich) at dilution of a 1:400, mouse anti-GFAP (BD Bioscience, #556328) at dilution of 1:100, mouse anti-NeuN (Millipore, #MAB377) at dilution of 1:100 at 4 °C overnight. After washing, slices were incubated with Alexa Fluor 594-conjugated Goat anti-rabbit secondary antibody (Thermo Fisher Scientific) or Alexa Fluor 488-conjugated Goat anti-mouse secondary antibody (Thermo Fisher Scientific). Fluorescence signals were captured with a Leica TCSSPE confocal Microscope and analysed with Fiji software.

### GST-L-DL co-affinity precipitation (co-AP)

293T cells were transfected with GST-control (GST-CTRL) or GST-L-DL plasmids and cell lysates were collected with GST-binding buffer (PBS buffer with 0.1 mM EDTA, 0.1% Triton X-100 and 1% proteinase inhibitor cocktails, pH 7.4). Cells then were sonicated until the lysate was clear and transparent. Protein concentration was measured with BCA Protein Assay Kit (Thermo Fisher Scientific, #23225) and 500 μg of cell lysates were used to incubate with 30 μl GST agarose beads (Cytiva, #17075601) overnight at 4 °C. Beads were washed 3 times with buffer A (PBS buffer with 0.1% SDS and 0.3% deoxycholate and 0.3% NP-40, pH 7.4) and buffer B (50 mM Tris-HCl with 10 mM MgCl_2_ and 0.5% NP-40, pH 7.4). GST-binding proteins were eluted with protein loading buffer (10 mM Tris-HCl with 0.4% SDS and 20 mM DTT and 2% glycerol and 0.05% bromophenol blue dye, pH 6.8). Beads were removed by centrifugation at 5,000 rpm for 1 min. The supernatant was collected for western blot or silver staining assay.

### Silver staining and Mass Spectrometry

Silver staining was performed as described previously [28]. Briefly, gels were submerged in fixative solution (50% ethanol, 12% acetic acid and 0.05% formalin) for 2 h, followed by washing with 20% EtOH twice for 40 min. 0.02% sodium thiosulfate solution was used to sensitize the gel for 2 min, followed by wash with deionized water. Gels were then incubated with 0.2% AgNO_3_ containing 0.076% formalin for 20 min, followed by wash with deionized water twice. Finally, gels were developed in 6% Na_2_CO_3_ solution containing 0.0004% Na_2_S_2_O_3_ and 0.05% formalin for 2–5 min and developing was terminated in 12% acetic acids. Target bands were subjected to Mass Spectrometry (MS) analysis (PTM Biolab). Proteins identified from MS are listed on Table S4. Functional annotations were performed on website: http://metascape.org/, with protein accession as previously described [29]. Cellular component enrichment analysis was conducted by a tool available on website: http://geneontology.org/. Analyses on L-DL-associated protein interactions were carried out on STRING database (https://string-db.org/) (v.11.0, *H. sapiens* dataset, PPI enrichment *P-*value: < 1.0e-16) according to previously published [30].

### Splicing reporter assay

Splicing reporter assay was conducted according to procedures previously described [24]. Briefly, L-DL KO and control (CTRL) N2a cells were transfected with luciferase-WT or luciferase-Mut for 12 h. The relative luciferase activity was calculated based on the following formula:

$$ \mathrm{Relative}\ \mathrm{luciferase}\ \mathrm{activity}=\frac{\mathrm{luciferase}\hbox{-} {\mathrm{Mut}}_{\mathrm{L}\hbox{-} \mathrm{DL}\ \mathrm{KO}}/\mathrm{luciferase}\hbox{-} {\mathrm{WT}}_{\mathrm{L}\hbox{-} \mathrm{DL}\ \mathrm{KO}}}{\mathrm{luciferase}\hbox{-} {\mathrm{Mut}}_{\mathrm{CTRL}}/\mathrm{luciferase}\hbox{-} {\mathrm{WT}}_{\mathrm{CTRL}}}. $$

### RNA extraction, reverse transcription and PCR for splicing analysis

Total RNA was extracted from cells or tissues using TRIzol Reagent (Invitrogen, #15596026), according to manufacturer’s protocol. RNA was reverse transcribed into cDNA using HiScriptIII 1st Strand cDNA Synthesis Kit (Vazyme, #R312). For splicing analysis, specific primers were used to amplify gene splicing isoforms using Easy Taq DNA Polymerase (Transgen, #AP111). The PCR products were analysed by  3% agarose TBE gel. The qualification of PCR products was conducted by Fiji software. Specific splicing primers are listed in Table S5.

### Next generation sequencing and alternative splicing analysis

For RNA-Sequencing (RNA-Seq), total RNA was isolated from cells and mRNA was enriched by Oligo (dT) beads, followed by fragmentation and reverse transcription with random primers. Obtained cDNAs were purified, their 5′ and 3′ ends were repaired and ligated with adapters. Ligated cDNAs were amplified by PCR and subjected to Illumina Novaseq system for 150 nt pair-end sequencing (Annoroad). Reads were aligned to the mouse genome (Mus_musculus.GRCm38.90.chr). For alternative splicing analysis, “percent spliced in” (PSI) values, computed via rMATS (v4.1.0) software, were used to define alternative splicing levels of exon skipping, intron retention, mutually exclusive exon inclusions, alternative 5′ splice sites, and alternative 3′ splice sites as previously reported [31]. The positive change of ΔPSI (PSI_L-DL KD_-PSI_CTRL_ > 0) represents increase of inclusion and negative change ΔPSI (PSI_L-DL KD_-PSI_CTRL_ < 0) represents decrease of inclusion.

### Generation of adeno-associated virus and hippocampal injection

Adeno-associated virus (AAV) was produced according to procedures previously reported [32, 33]. In Brief, AAV plasmid with target sequences, pHelper and helper 2/9 plasmid were co-transfected into 293T cells in a ratio of 2:1:1 by PEI. Twenty-four hours after transfection, medium was changed to DMEM plus 2% FBS. Both cells and medium were collected for AAV purification 72 h after transfection.

AAV particles were released from cells with freeze/thaw cycles, followed by treatment with 50 U/ml benzonase nuclease (MKbio) and 10 U/ml RNase I (Vazyme) at 37 °C for 30 min, and incubation for another 30 min upon adding of 0.5% sodium deoxycholate (Sigma-Aldrich). Cell debris were removed by centrifugation at 2,500 g for 30 min, 40% PEG8000 and 2.5 M NaCl were added to precipitate the virus. The viral pellet was re-suspended in PBS, and contaminated proteins were removed by chloroform and (NH_4_)_2_SO_4_ extraction. Viral titter was determined by qPCR-based approach.

Male wild-type C57BL/6 J mice at 8 weeks of age were stereotaxically injected with L-DL shRNA (0.8 μl, 2 × 10^11^ TU/ml) or control shRNA (0.8 μl, 8 × 10^11^ TU/ml) adenovirus into the bilateral CA1 areas of hippocampus with an air pressure injector system (KDS). Male APP/PS1 mice at 6 months of age were stereotaxically injected with L-DL shRNA (0.8 μl, 2 × 10^11^ TU/ml) or control shRNA (0.8 μl, 8 × 10^11^ TU/ml) adenovirus, with L-DL overexpression (0.5 μl, 7 × 10^11^ TU/ml) or control adenovirus (0.5 μl, 8 × 10^11^ TU/ml) into the bilateral CA1 areas of hippocampus with an air pressure injector system (KDS). The coordinates used for stereotaxic injections were AP -2.3, ML 2.0, DV -1.5 and AP -2.3, ML -2.0, DV − -1.5. Behavioural tests were conducted 4 weeks after the injection.

### Behavioural assays

#### The Morris water maze

To evaluate spatial learning and memory, the Morris water maze task was performed according to previously published [34]. Briefly, a platform (10 cm) was submerged in a black circular pool (diameter: 120 cm). During the training phase, each individual mouse received consecutive trials for continuous 5 days. On probe trial day, the platform was removed and mice were allowed to swim for 90 s starting from site opposite to where the platform was located. Behavioural parameters were recorded by a video camera set on top of the circular pool and data were analyzed using Ethovision XT 11 software (Noldus).

#### Novel object recognition (NOR)

The Novel object recognition task was performed as described previously, with modifications [35, 36]. Briefly, all mice were placed in an open field box, and allowed to freely explore the empty open field arena for 5 min on day 1. On day 2, each mouse was subjected to familiarization phase with exploration of two identical objects (A + A) for 5 min. 4 h after the familiarization phase, mouse was allowed to explore the field with a familiar object (A) and a novel object (B) at the same position for 5 min. Behavioural parameters were recorded by a video camera set on top of the arena  and data were analyzed using Ethovision XT 11 software (Noldus). The discrimination index is calculated as the ratio of time to explore the novel object to total exploring time on both objects.

#### Y maze

Modified version of Y maze test was performed as described previously, with modifications, in white polypropylene walls with three arms (10 × 40 × 16 cm) [37, 38]. This test comprises a sample phase trial and a test phase trial. In the sample phase trial, mice were placed in the maze with one of 3 arms closed, and allowed to freely explore for 6 min. Chambers were cleaned with 70% ethanol before and after each use. 6 h after the sample phase trial, mice were allowed to freely explore the field of maze with 3 arms opened for 6 min in the test phase trial. The arm previously closed in the sample phase trial was defined as the novel arm. Behavioural parameters were recorded by a video camera and time spent in novel arm were analyzed using Ethovision XT 11 software (Noldus).

#### Open field

The Open field task was performed as described previously in a large square chamber [39]. During the test, mice were placed in a corner square with its head facing the corner of the open field apparatus, and were allowed to explore for 8 min to record its exploratory activities. Chambers were cleaned with 70% ethanol before and after each use. Behavioural parameters were recorded by a video camera and data were analyzed using Ethovision XT 11 software (Noldus).

#### Quantification and statistical analysis

All quantified data represent an average of at least triplicate samples. Error bars represent standard error of the mean. Statistical significance was determined by Student’s *t*-test or two-way ANOVA in GraphPad Prism 5.0. *P* < 0.05 was considered significant (indicated by an asterisk in the figures), *P* < 0.01 (indicated by two asterisks in the figures), *P* < 0.001 (indicated by three asterisks in the figures), n.s. not significant.

## Results

### L-DL shows an age-dependent decrease in the brain

**Fig. 1:**
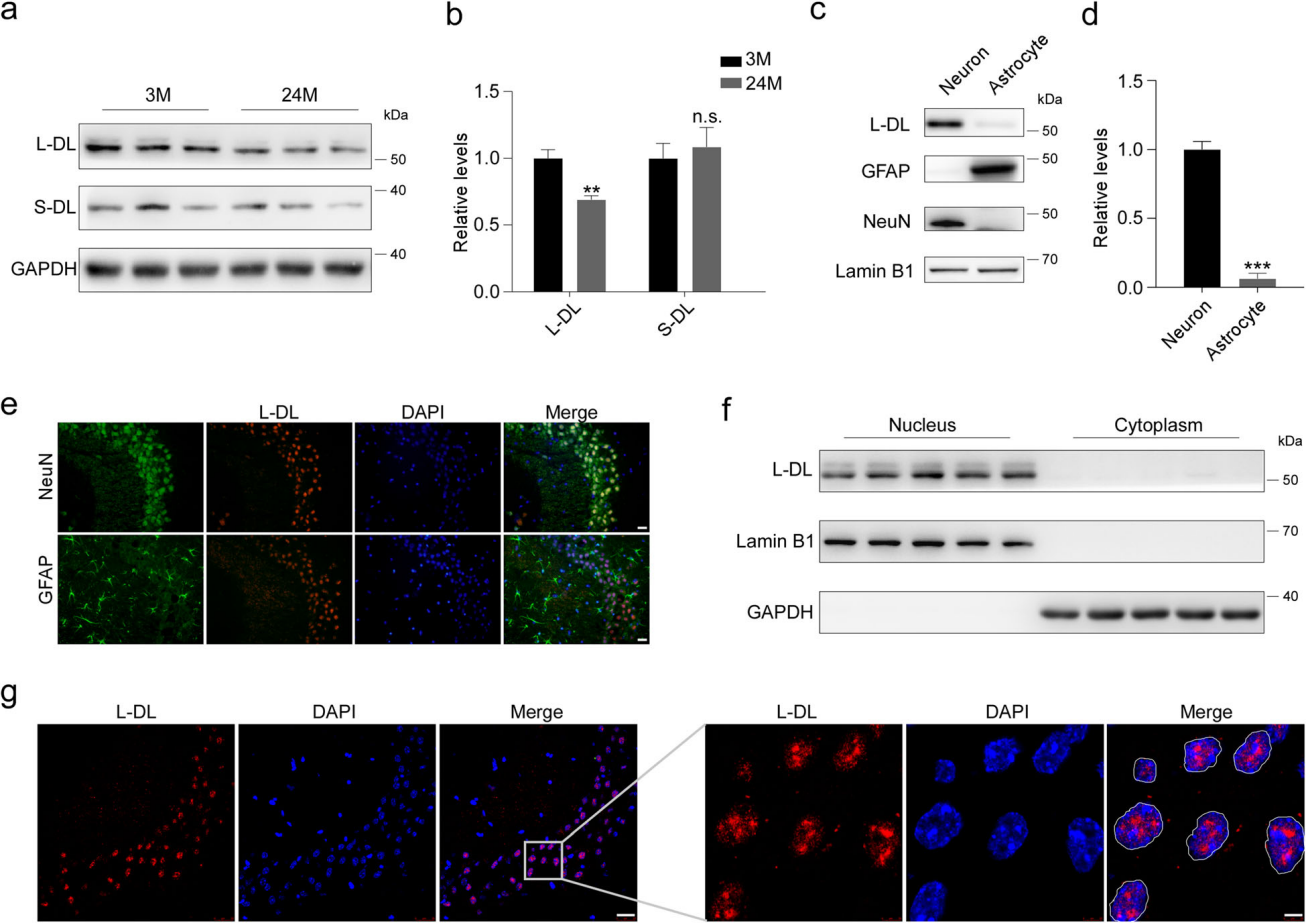
L-DL shows an age-dependent decrease in the brain. **a**, **b** Levels of L-DL and S-DL in the hippocampus of 3-month- and 24-month-old mice (3 M and 24 M, respectively; *n* = 3 per group), determined by immunoblotting (**a**) and densitometric analyses (**b**). GAPDH was included as an input control. **c**, **d** Levels of L-DL, NeuN, a neuronal marker, and GFAP, an astrocytic marker, in cultured neurons and astrocytes, were determined by immunoblotting (**c**) and densitometric analyses (*n* = 3) (**d**). **e** Representative immunofluorescence images of NeuN or GFAP (green), L-DL (red) and DAPI (blue) in the CA1 of hippocampus of 3-month-old (3 M) mice. Scale bars: 10 μm. **f** Protein levels of L-DL in the nucleus and cytoplasm of hippocampal tissue lysates, as measured by immunoblotting. Lamin B1 and GAPDH were included as input controls. **g** Representative immunofluorescence images of L-DL (red) and DAPI (blue) in the CA1 region of 3-month-old (3 M) mice. The right panel showed magnifications of grey squares indicated in the overview images of the left panel. The nucleus is denoted by white circles. Scale bars: 25 μm. Statistical analysis was performed using by ANOVA or two-tailed Student’s *t*-test; ** *P* < 0.01, *** *P* < 0.001; error bars denote SEM

hnRNP DL (DL) is a highly conserved nuclear RNA binding protein located in the genomic position 4q21 [40]. Two DL transcripts have been identified previously [41], which are translated into long- and short-isoform of DL proteins, namely L-DL and S-DL, with molecular weights of 53 kDa and 38 kDa respectively (Fig. S1a). To investigate their expression patterns upon aging progression, we assessed the levels of L-DL and S-DL in the hippocampus of 3-month-old mice (young) and 24-month-old mice (aged). We found a significant reduction of L-DL, but not S-DL, in aged mouse hippocampus (Fig. 1a, b). Moreover, L-DL was preferentially expressed in neurons and its expression was almost undetectable in astrocytes (Fig. 1c, d). This neuronal localization of L-DL was also confirmed by immunostaining (Fig. 1e). Notably, L-DL was predominantly localized to the nucleus, as demonstrated by subcellular fractionation and immunostaining (Fig. 1f, g). These findings suggest that L-DL is predominantly localized to neuronal nuclei and displays an age-dependent reduction in the hippocampus of mouse brain.

### Loss of L-DL in the hippocampus leads to cognitive decline in mice

**Fig. 2:**
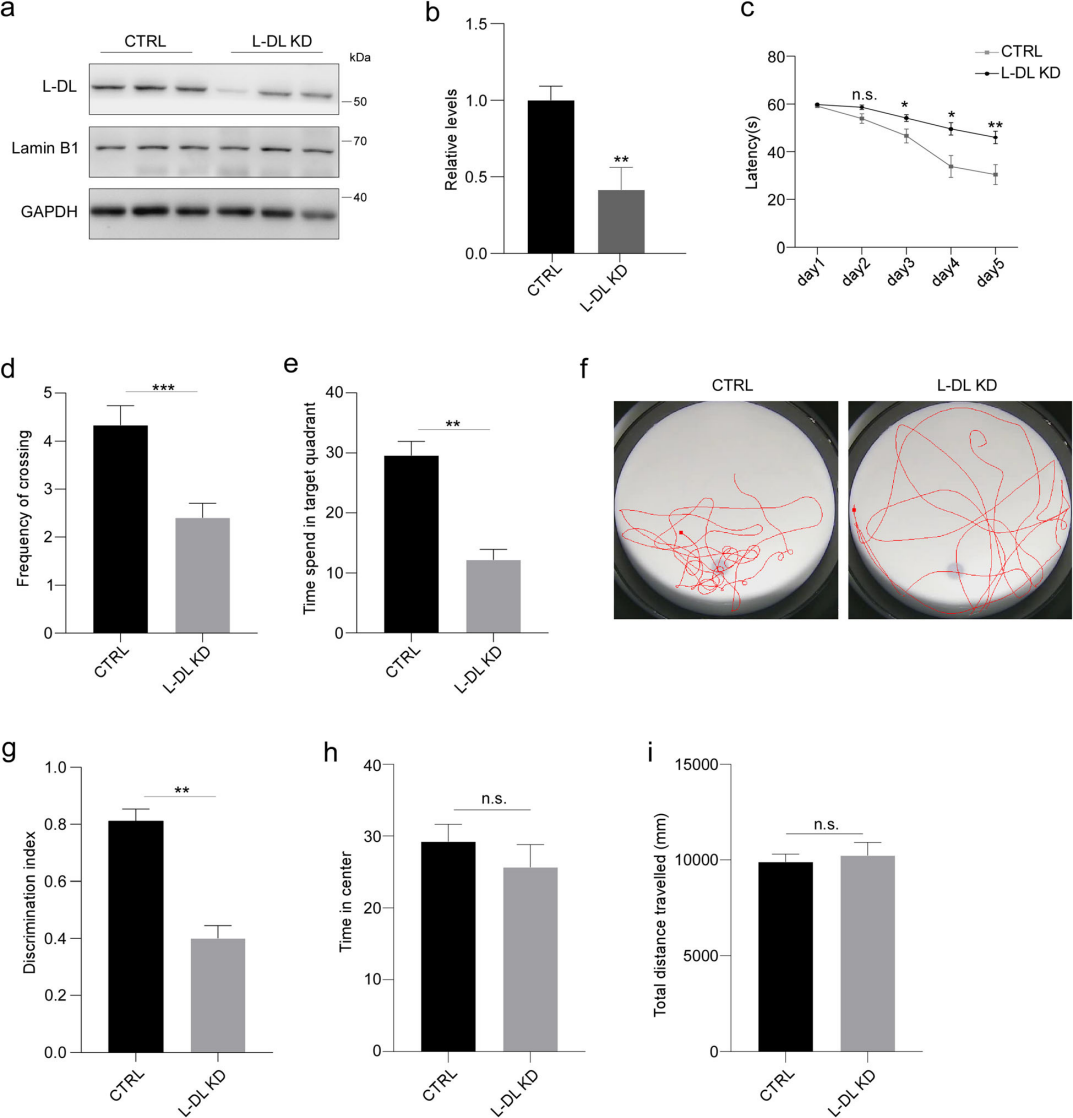
Loss of L-DL in the hippocampus leads to cognitive deficits in mice. **a**, **b** L-DL knockdown efficiency in the hippocampus, as determined by immunoblotting (**a**) and densitometric analyses in (**b**). **c**-**f** The Morris water maze task. **c** Time of escape latency to the hidden platform was plotted for each spatial learning day (*n* = 15 mice per group). The frequency of crossing the target quadrant (**d**) and time spent in the target quadrant (**e**) in the probe trial (n = 15 mice per group). **f** The representative escape routes for control and L-DL KD mice in the probe trial. **g** Discrimination index for the object recognition test (*n* = 15 mice per group). **h**, **i** In the open field task, time spend in the center of the compartment (**h**) and total distance travelled (**i**) for control and L-DL KD mice (*n* = 15 mice per group). Statistical analysis was performed using by ANOVA or two-tailed Student’s *t*-test; * *P* < 0.05, ** *P* < 0.01, *** *P* < 0.001; error bars denote SEM

To determine the function of L-DL in cognition, we next conducted L-DL knockdown (KD) in the CA1 region of hippocampus of 8 weeks-old C57BL/6 J wild-type (WT) mice, using AAV delivered shRNA technology. We injected multiple L-DL shRNA sequences into the hippocampus of WT mouse brains, and KD efficiency was assessed by examining the levels of L-DL in the injected brains (Fig. 2a, b and Fig. S1b, c). We next conducted a series of behavioural tests to assess the cognitive function of these mice. In the Morris water maze task, mice with reduced L-DL expression spent significantly longer time to find the hidden platform comparing to control mice during the training phase (Fig. 2c). In the probe trial, these mice also showed fewer platform crossings and spent less time in the target quadrant (Fig. 2d-f). In the novel object recognition task, L-DL KD mice spent less time to explore the novel objects (Fig. 2g). These results indicate that L-DL KD mice show defective spatial and contextual memory. Notably, L-DL KD and control mice exhibited similar spontaneous locomotor activity and anxiety-like behaviours, as indicated by the open field task, ruling out the effect of locomotor activity and anxiety on the readout of the Morris water maze test (Fig. 2h, i). Taken together, these findings demonstrate that L-DL is tightly associated to cognitive function in mice.

### L-DL is an essential component of nuclear speckle

**Fig. 3:**
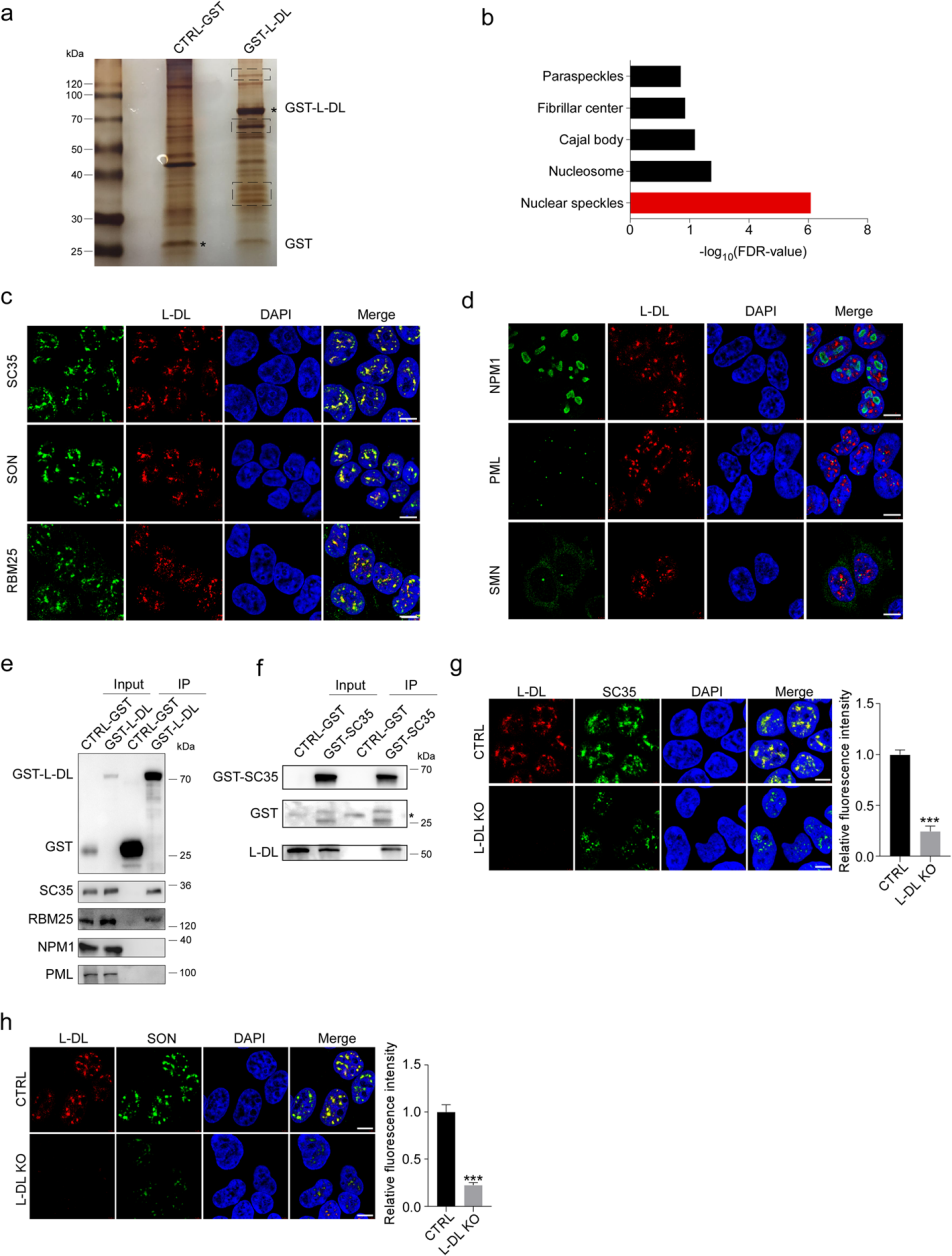
L-DL is an essential structural component of nuclear speckle. **a** Control GST (CTRL-GST) and GST tagged L-DL (GST-L-DL) were introduced into 293T cells, followed by pull-down with GST agarose beads. L-DL associated proteins were subjected to silver staining, black square denoted bands were subjected to mass spectrometry analysis. **b** Cellular component terms in the gene ontology (GO) analysis for L-DL associated proteins identified in mass spectrometry. **c** Representative immunofluorescence images of L-DL (red) and SC35 (green), L-DL (red) and SON (green), L-DL (red) and RBM25 (green), and DAPI (blue) in 293T cells. Scale bars: 10 μm. **d** Representative immunofluorescence images of L-DL (red) and NPM1 (green), L-DL (red) and PML (green), L-DL (red) and SMN (green), and DAPI (blue) in 293T cells. Scale bars: 10 μm. **e** CTRL-GST and GST-L-DL were introduced into 293T cells, followed by pull-down with GST agarose beads. L-DL associated proteins were subjected to immunoblotting with anti-SC35, anti-RBM25, anti-NPM1 and anti-PML antibodies. **f** CTRL-GST and GST-SC35 were introduced into 293T cells, followed by pull-down with GST agarose beads. SC35 associated proteins were subjected to immunoblotting with anti-L-DL antibody. **g** Left: representative immunofluorescence images of L-DL (red), SC35 (green) and DAPI (blue) in control and L-DL knockout (KO) 293T cells. Scale bars: 10 μm. Right: Densitometric analyses of SC35 fluorescence density. **h** Left: representative immunofluorescence images of L-DL (red), SON (green) and DAPI (blue) in control and L-DL KO 293T cells. Scale bars: 10 μm. Right: densitometric analyses of SON fluorescence density. Statistical analysis was performed using two-tailed Student’s *t*-test; *** *P* < 0.001; error bars denote SEM

To identify L-DL binding proteins, we next performed affinity-precipitation (AP), followed by SDS-PAGE and mass spectrometry (MS) (Fig. 3a). Intriguingly, gene ontology (GO) analyses showed that L-DL associated proteins are mostly nuclear speckle proteins (Fig. 3b). We therefore examined the subcellular localization of L-DL and found that L-DL was co-localized with nuclear speckle markers SC35, SON and RBM25, demonstrating that L-DL is specifically localized to nuclear speckles (Fig. 3c). No associations of L-DL with NPM1, a nucleolar protein; with promyelocytic leukemia protein (PML), a PML body marker; and with SMN, a Cajal body marker, were detected by immunostaining, suggesting that L-DL was not localized to those nuclear bodies (Fig. 3d). GST-pulldown and co-AP assays confirmed the association of L-DL with the speckle component proteins SC35 and RBM25 (Fig. 3e, f). Our findings demonstrate that L-DL is preferentially localized to nuclear speckles. We also examined the subcellular localization of other hnRNP members by immunostaining and found that hnRNP A1, hnRNP C, hnRNP K and hnRNP U showed homogeneous distribution within the nucleus (Fig. S2a).

Interestingly, L-DL knockout (KO) resulted in cellular expression pattern changes of SC35 and SON from densely packed to diffusely distributed, suggesting that L-DL deficiency leads to disruption of nuclear speckle structure (Fig. 3g, h). These findings indicate that L-DL is an essential component and is important for maintaining the integrity of nuclear speckles by interacting with other key speckle proteins such as SC35 and SON.

### Downregulation of L-DL induces alternative splicing changes in the brain

**Fig. 4:**
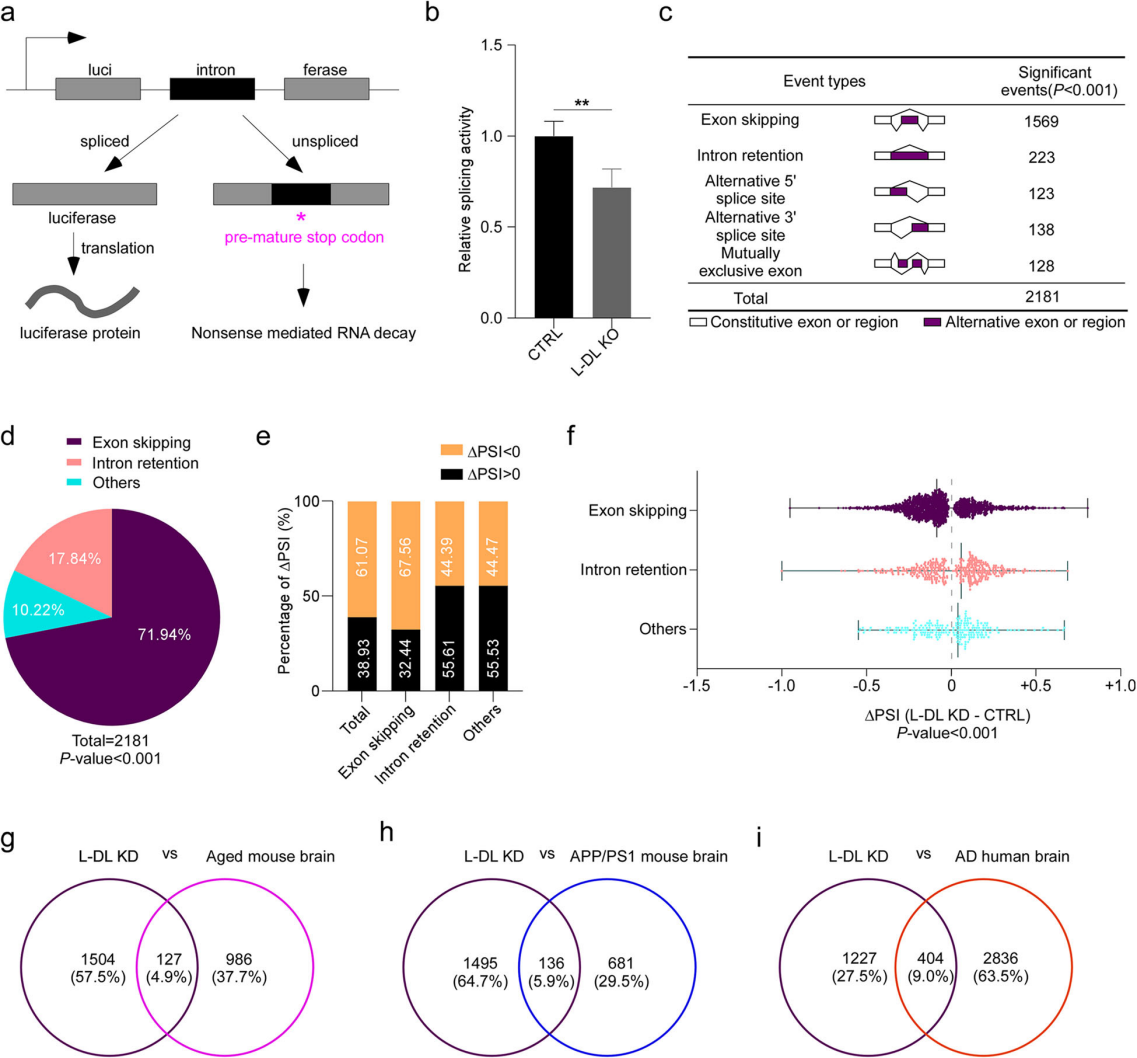
Lack of L-DL results in alternative splicing changes in the brain. **a** Diagram for the splicing reporter used to measure splicing efficiency. **b** Luciferase-WT or luciferase-Mut were introduced into control or L-DL KO N2a cells. Luciferase levels were plotted as fold differences of L-DL KO over controls (*n* = 3). **c** Number of significantly changed alternative splicing events in L-DL knockdown (KD) hippocampus. **d** Pie chart indicating the distribution of exon skipping, intron retention and other splicing events such as alternative 5′ splice site, alternative 3′ splice site and mutually exclusive exon in the hippocampus of L-DL KD mice. **e** Distribution of positive ΔPSI (PSI_L-DL KD_-PSI_CTRL_ > 0) and negative ΔPSI (PSI_L-DL KD_-PSI_CTRL_ < 0) for total changed splicing events (*n* = 2,181), exon skipping (*n* = 1,569), intron retention (*n* = 223) and other splicing events (*n* = 389) (*P*-value< 0.001). **f** Distribution of ΔPSI values (PSI_L-DL KD_-PSI_CTRL_) in splicing events such as exon skipping (*n* = 1,569), intron retention (*n* = 223) and other splicing events (*n* = 389) (*P*-value < 0.001). Data were shown as median within range. **g**-**i** Venn diagram demonstrating the intersections of genes with changed alternative splicing that identified in L-DL KD mouse brains and differentially expressed genes (DEGs) between aged and WT mouse brains (**g**), of genes with changed alternative splicing in L-DL KD mouse brains and DEGs between APP/PS1 and WT mice (**h**), of genes with changed alternative splicing in L-DL KD mouse brains and DEGs between AD patients and age-matched controls (**i**). Statistical analysis was performed using two-tailed Student’s *t*-test; ** *P* < 0.01; error bars denote SEM

Nuclear speckle is highly enriched with RNA processing factors and its functions are predominantly linked to pre-mRNA splicing [42]. The nuclear speckle localization of L-DL prompted us to investigate the function of L-DL in RNA splicing. We next assessed the splicing activity by using an intron-containing luciferase reporter and found a significant reduction in splicing activity in L-DL depleted N2a cells (KO) [24] (Fig. 4a, b), suggesting that L-DL participates in RNA splicing process.

We next investigated whether L-DL regulates cognition through modulating RNA splicing. Hippocampal tissues from control and L-DL KD mice were subjected to RNA sequencing and subsequent splicing analyses. Splicing analyses demonstrated that a total of 2,181 splicing events displayed significant splicing pattern changes in L-DL KD brains, wherein exon skipping was the most dominant event (71.94%); other splicing events included intron retention (10.22%), mutually exclusive exon inclusions, retained introns, alternative 5′ splice sites, and alternative 3′ splice sites (17.84%) (Fig. 4c-d). We used “percent spliced in” (PSI) values to define the alternative splicing levels of each gene segment by the rMATs software as described previously [31]. We found that 38.93% of these gene segments showed increased inclusion (PSI_L-DL KD_-PSI_CTRL_ > 0, *P* < 0.001) and 61.07% showed increased exclusion (PSI_L-DL KD_-PSI_CTRL_ < 0, *P* < 0.001) in L-DL KD brains (Fig. 4e-f). Of note, segment exclusion in L-DL KD brains occurred more often in exon skipping, compared to intron retention and other splicing events. Conversely, segment inclusion occurred more often in intron retention and other splicing events than exon skipping in L-DL KD brains (Fig. 4e-f). All these findings demonstrate that L-DL deficiency leads to significant changes in multiple alternative splicing events in the brain.

A substantial change in gene expression has been observed in the brain of aged mice [43] and AD model mice (APP/PS1) [44, 45]. To ascertain the relationship between the previously identified differentially expressed genes (DEGs) in aged vs young or AD vs control brains and genes with altered alternative splicing patterns identified in the L-DL KD mouse brain, we next conducted overlapping analysis: DEGs were pulled out from (1) RNA-seq data of young vs old mouse brains (GSE129788), (2) RNA-seq data of APP/PS1 vs WT mouse brains (GSE132177), and (3) proteomic data of human AD brains [45]. We found that a total of 127 genes (4.9%) corresponding to intersections of genes with changed alternative splicing pattern in L-DL KD mouse brains and DEGs in aged vs young mouse brains (Fig. 4g). A total of 136 genes (5.9%) corresponding to intersections of genes with changed alternative splicing pattern in L-DL KD mouse brains and DEGs in AD vs WT mouse brains (Fig. 4h). Importantly, we uncovered a total of 404 genes (9%) corresponding to intersections of genes with changed alternative splicing pattern in L-DL KD mouse brains and DEGs in AD vs control human brains (Fig. 4i). These findings demonstrate that L-DL downregulation-induced splicing changes likely contribute to aging and age-related diseases.

### L-DL deficiency impairs U2 spliceosome-dependent RNA splicing

**Fig. 5:**
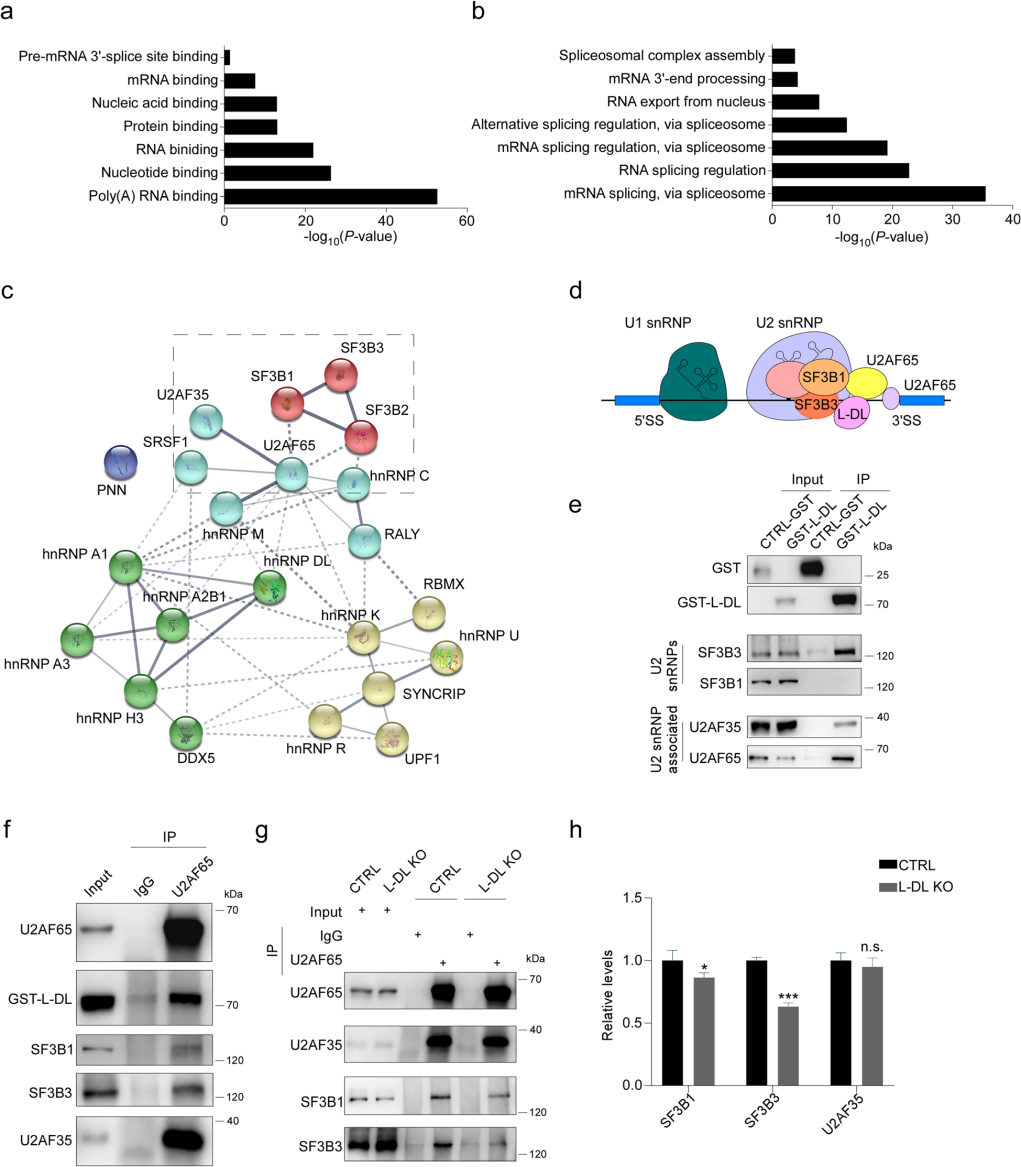
L-DL deficiency impairs U2 spliceosome dependent RNA splicing. (**a**-**b**) Molecular function (**a**) and biological process (**b**) terms in GO analysis for L-DL associated proteins identified in mass spectrometry. **c** Protein-protein interaction (PPI) network for L-DL associated proteins (PPI enrichment *P* < 1.0E-16). The network clustering is based on k-mean clustering and each cluster is labelled with a distinct colour. **d** A drawing of U2 spliceosome assembly on pre-mRNAs. 5′SS or 3′SS indicate 5′ or 3′ splicing sites, respectively. **e** CTRL-GST or GST-L-DL were introduced into 293T cells, followed by pull-down with GST agarose beads. L-DL associated proteins were determined by immunoblotting. **f** GST-L-DL was introduced into 293T cells, followed by immunoprecipitation with anti-U2AF65 antibody. Levels of U2AF65 associated proteins were measured by immunoblotting. **g**, **h** Control or L-DL KO N2a cell lysates were subjected to immunoprecipitation with anti-U2AF65 antibody. Levels of U2AF65 associated proteins were measured by immunoblotting (**g**) and densitometric analyses (*n* = 3) (**h**). Statistical analysis was performed using two-tailed Student’s *t*-test; * *P* < 0.05, ** *** *P* < 0.001; error bars denote SEM

GO analyses showed that L-DL associated proteins identified in MS exhibited an enriched functional annotation related to RNA binding and RNA splicing process (Fig. 5a, b). Network analysis on L-DL associated proteins enriched in “mRNA splicing via spliceosome” term indicated that L-DL interacts with multiple components of U2 spliceosome complex (Fig. 5c). U2 snRNP is a core component of the spliceosome, and SF3B is a core component of U2 snRNP [46]. U2AF is required for U2 snRNP binding and splicing complex assembly [3, 47], wherein U2AF35 binds to the 3′ splice sites and U2AF65 binds to the polypyrimidine tract of pre-mRNAs (Fig. 5d). The association of L-DL and U2 snRNPs was validated by Co-AP (Fig. 5e). In particular, L-DL was found to associate with U2AF35, U2AF65 and SF3B3, but not with SF3B1 (Fig. 5e). The association of L-DL and U2AF65 was further confirmed by Co-IP (Fig. 5f). In addition, both SF3B1 and SF3B3 are also associated with U2AF65 (Fig. 5f). Importantly, L-DL deficiency substantially reduced the association of U2AF65 with SF3B1 and SF3B3 (Fig. 5g, h). These findings indicate that L-DL is an integral and essential component of U2 snRNP, and loss of L-DL may disrupt loading of U2 snRNP on pre-mRNAs. Notably, we also identified multiple hnRNPs, such as hnRNP A1, hnRNP C, hnRNP U and hnRNP K as L-DL associated proteins in MS (Fig. 5c). Given that these identified hnRNPs are reportedly involved in RNA splicing process [48], we next conducted Co-AP to validate the association of L-DL and these hnRNPs. We found that L-DL indeed was associated with hnRNP A1, hnRNP C, hnRNP U and hnRNP K, further supporting the involvement of L-DL in RNA splicing process (Fig. S2b).

### L-DL regulates alternative splicing of genes involved in synaptic function

**Fig. 6:**
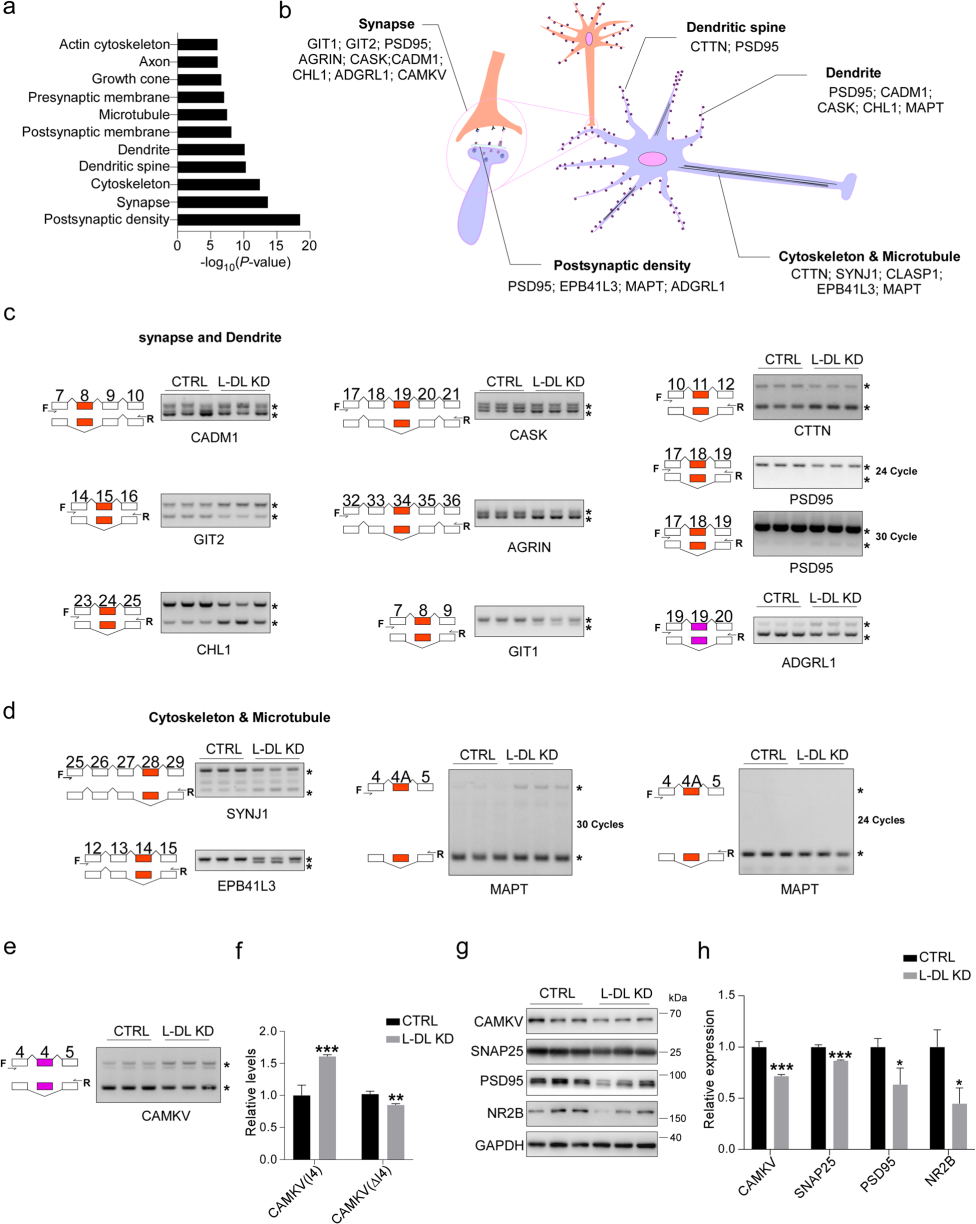
L-DL regulates cytoskeleton- and synapse-related gene expressions via modulating their alternative splicing. **a** Cellular component terms for genes with changed alternative splicing in the hippocampus of L-DL knockdown (KD) mice in GO analysis (*n* = 1,631, *P* < 0.001). **b** Diagram for cellular localization of synapse-, dendrite- and cytoskeleton-related proteins, with their genes undergoing  aberrant alternative splicing in neurons. **c**, **d** Alternative splicing products of CADM1, GIT2, CHL1, CASK, AGRIN, GIT1, CTTN, PSD95, ADGRL1, SYNJ1, EPB41L3 and MAPT gene, determined by RT-PCR in the hippocampus of control and L-DL KD mice (*n* = 3). * indicates spliced variants, F and R denote primers used for RT-PCR. 24 cycles or 30 cycles were used to detect higher abundant and lower abundant splicing products, respectively. **e**, **f** Alternative splicing products of CAMKV determined by RT-PCR (**e**) and densitometric analyses (**f**) in the hippocampus of control and L-DL KD mice (n = 3). I4, intron 4 retention; ΔI4, intron 4 exclusion. **g**, **h** Protein levels of CAMKV, SNAP25, PSD95, NR2B in the hippocampus of control and L-DL KD mice, measured by immunoblotting (**g**) and densitometric analyses (**h**). GAPDH was included as an input control. Statistical analysis was performed using two-tailed Student’s *t*-test; * *P* < 0.05, ** *P* < 0.01, *** *P* < 0.001; error bars denote the SEM

Given that a loss of L-DL leads to cognitive decline in WT mice (Fig. 2c-g), we postulated that L-DL regulates cognitive function through modulating alternative splicing and expression of genes that are involved in synaptic function. Indeed, KEGG pathway and GO analyses revealed significant changes in alternative splicing of genes that were closely related to synaptic function and cognitive process (Fig. S3a-c). In addition, cellular component analyses revealed significant changes in alternative splicing of genes related to multiple cellular structures of neuronal cells, including postsynaptic density, synapse, cytoskeleton and dendritic spine (Fig. 6a). Some of well-defined gene-encoded productss and their cellular localizations were illustrated in Fig. 6b, e.g., dendrite and synapse related genes such as cell adhesion molecule 1 (CADM1), which is a synaptic cell adhesion molecule that regulates synaptogenesis [49]; G protein-coupled receptor kinase interacting protein 2 (GIT2), which regulates cytoskeletal structure and presynaptic neurotransmitter release [50, 51]; cell adhesion molecule L1 like (CHL1), which is accumulated in presynaptic membranes and regulates synaptic activity and plasticity [52]; calcium/calmodulin-dependent serine protein kinase (CASK), which is a synapse scaffolding protein that is involved in synapse formation and function [53]; AGRIN, a heparan sulfate proteoglycan that promotes the formation of excitatory synapses [54]; G protein-coupled receptor kinase interacting protein 1 (GIT1), which regulates microtubule assembly and promotes synapse formation and maintenance [55]; Src Substrate Cortactin (CTTN), which is involved in actin polymerization and activity-dependent synaptic plasticity [56]; PSD95, a postsynaptic scaffolding protein that is required for activity-driven synapse stabilization [57]; Adhesion G protein-coupled receptor L1 (ADGRL1), which is involved in synapse formation and brain development [58]. In addition, some of the genes are highly related to cytoskeleton or microtubule formation, e.g., polyphosphoinositide phosphatase synaptojanin 1 (SYNJ1), which participates in actin cytoskeleton polymerization and synaptic vesicle recycling [59]; erythrocyte membrane protein band 4.1 like 3 (EPB41L3), which is related to actin binding and protein-protein interactions at synapses [60, 61]; microtubule-associated protein tau (MAPT) gene, which encodes tau protein that is involved in axonal transport, synaptic plasticity and function [62]. Of note, hippocampus-dependent memory is tightly associated with microtubules dynamics [63].

We next verified the alternative splicing patterns of dendrite and synapse related genes, and observed significant decreased skipping over exon 8 of CADM1, over exon 15 of GIT2, significant increased skipping over exon 24 of CHL1, over exon 19 of CASK, over exon 34 of AGRIN, over exon 8 of GIT1, over exon 11 of CTTN, and over exon 18 of PSD95 and significant increased intron retention over intron 19 of ADGRL1 (Fig. 6c). We also assessed the alternative splicing patterns of cytoskeleton-related genes, and found significant increased skipping over exon 28 of SYNJ1, over exon 14 of EPB41L3 and decreased skipping over exon 4A of MAPT (Fig. 6d). Importantly, calmodulin kinase-like vesicle-associated (CAMKV) gene, whose deficiency in CA1 pyramidal neurons impairs synaptic plasticity and spatial memory [64], showed a significant increase in intron 4 retention, leading to reduced production of CAMKV protein (Fig. 6e-h). We further found that the levels of synaptic proteins SNAP25, PSD95, and NMDA receptor subunit NR2B were significantly decreased in the hippocampus of L-DL KD mice (Fig. 6g, h). These findings strongly indicate that L-DL regulates cognition via modulating the alternative splicing of genes that are involved in synaptic function.

### L-DL improves cognitive function in AD mice

**Fig. 7:**
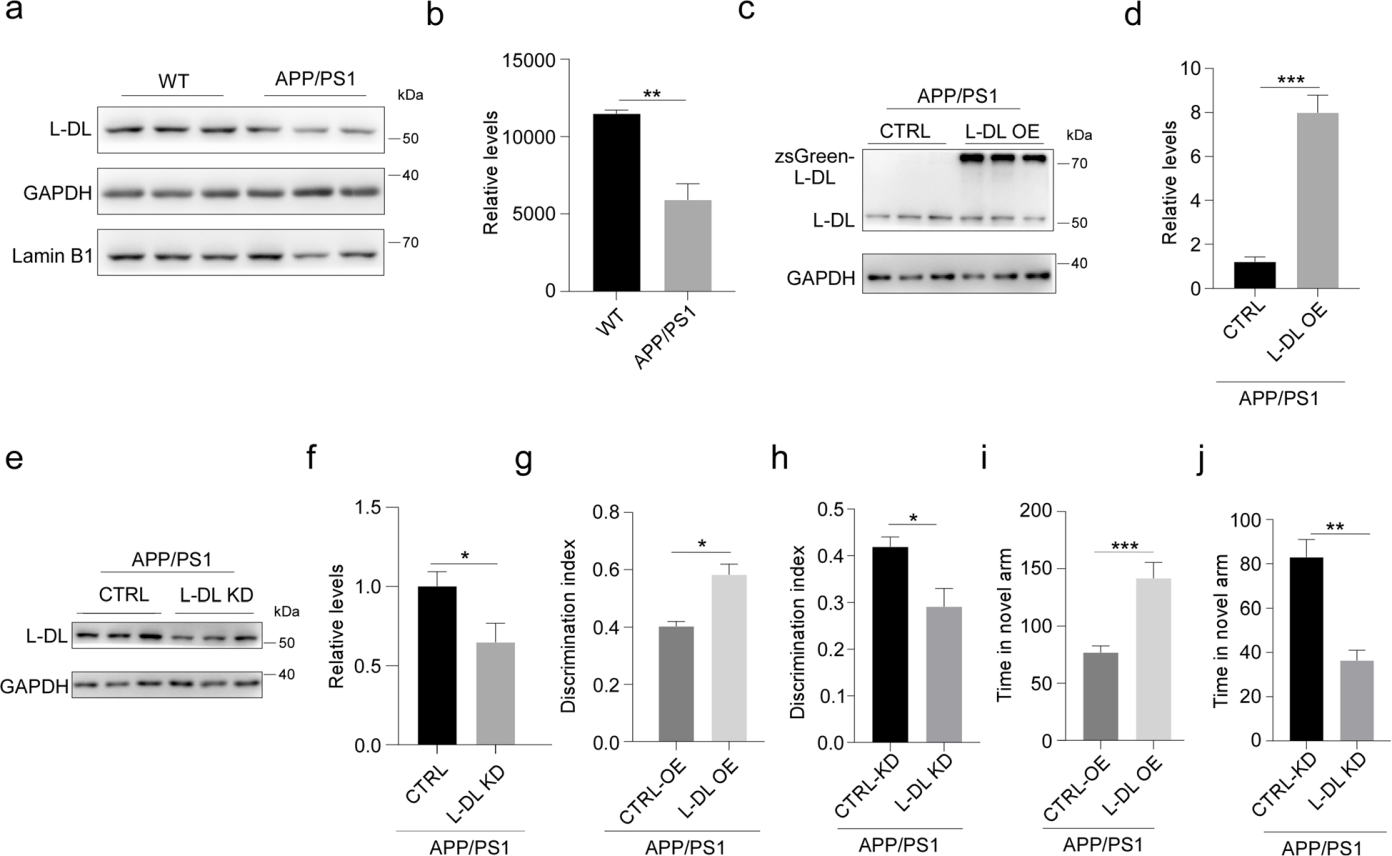
L-DL improves cognitive function in APP/ PS1 mice. **a**, **b** Levels of L-DL in the hippocampus of 6-month-old APP/PS1 and age-matched WT mice, determined by immunoblotting (**a**) and densiometric analysis (*n* = 3) (**b**). **c**, **d** Levels of L-DL in the hippocampus of APP/PS1 mice administered with zsGreen-L-DL, determined by immunoblotting (**c**) and densiometric analysis (*n* = 3) (**d**). **e**, **f** Levels of L-DL in the hippocampus of APP/PS1 mice treated with control or L-DL shRNA, determined by immunoblotting (**e**) and densiometric analysis (*n* = 3) (**f**). **g**-**j** APP/PS1 mice with L-DL overexpression (OE) or knockdown (KD) were subjected to behaviour assays. **g**, **h** Discrimination index for APP/PS1 mice with L-DL OE (**g**) or L-DL KD (**h**) in novel object recognition task (*n* = 15 mice per group). **i**, **j** Time spending in the novel arm for APP/PS1 mice with L-DL OE (**i**) or L-DL KD (**j**) in Y maze (*n* = 15 mice per group). Statistical analysis was performed using two-tailed Student’s *t*-test; * *P* < 0.05, ** *P* < 0.01, *** *P* < 0.001; error bars denote SEM

Given that L-DL is decreased in aged mouse brains and downregulation of L-DL impairs mouse memory, we next examined the potential role of L-DL in age-related neurodegenerative diseases, especially AD. We first observed a significant reduction of L-DL in the hippocampus of APP/PS1 mice (Fig. 7a, b), and therefore conducted L-DL overexpression (OE) or knockdown (KD) in the hippocampus of APP/PS1 mice. L-DL overexpression was driven by CamKII promoter, and L-DL KD was achieved by target shRNA expression driven by H1 promoter. AAV-mediated delivery was achieved via brain injection into the bilateral CA1 areas of the hippocampus in APP/PS1 mice at 6 months of age. L-DL expression was confirmed by immunoblotting 4 weeks after the injection (Fig. 7c-f). Behavioural assays showed that L-DL OE significantly improved contextual memory in APP/PS1 mice, as demonstrated by increased discrimination index in novel object recognition task; and increased time spending in the novel arm in Y maze task, compared to control APP/PS1 mice (Fig. 7g, i). L-DL OE did not affect locomotor activity and anxiety in APP/PS1 mice (Fig. S4a, c). Conversely, APP/PS1 mice with L-DL KD displayed deteriorated contextual memory, as demonstrated by decreased discrimination index in novel object recognition task and decreased time spending in novel arm in Y maze, compared to control APP/PS1 mice (Fig. 7h, j). APP/PS1 mice with L-DL KD showed no difference in their locomotor activity and anxiety, when compared to control mice (Fig. S4b, d).

### L-DL improves cognition in AD mice via modulating alternative splicing and expression of synaptic genes

**Fig. 8:**
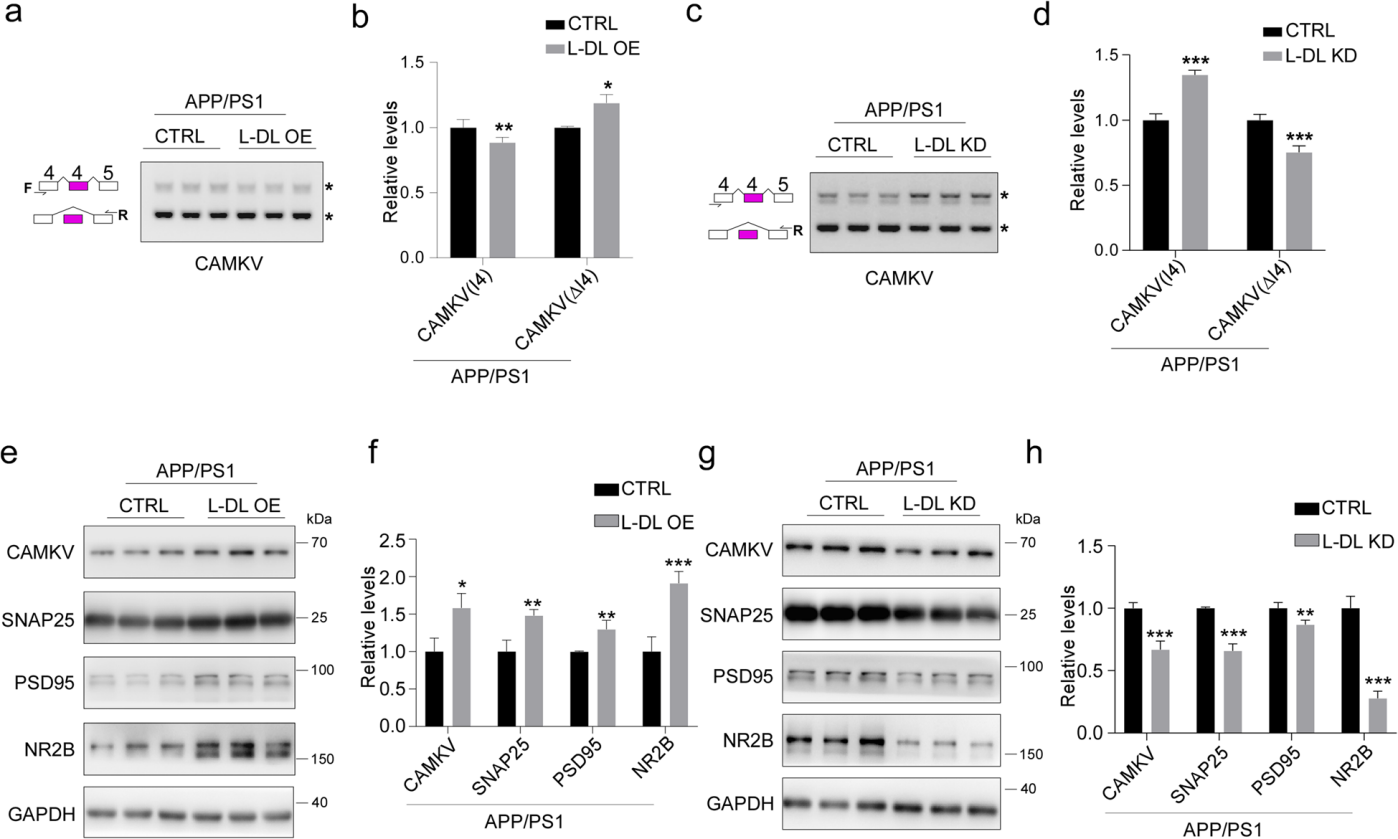
L-DL improves cognition in AD mice via modulating alternative splicing and expression of synaptic genes. **a**, **b** Alternative splicing products of CAMKV, determined by RT-PCR (**a**) and densitometric analyses (**b**) in the hippocampus of APP/PS1 mice with control or L-DL OE (n = 3 per group). **c**, **d** Alternative splicing products of CAMKV, determined by RT-PCR (**c**) and densitometric analyses (*n* = 3) (**d**) in the hippocampus of APP/PS1 mice with control and L-DL KD (*n* = 3 per group). **e**, **f** Protein levels of CAMKV, SNAP25, PSD95, NR2B in the hippocampus of APP/PS1 mice with control or L-DL OE (*n* = 3 per group), determined by immunoblotting (**e**) and densitometric analyses (**f**). GAPDH was included as an input control. **g**, **h** Protein levels of CAMKV, SNAP25, PSD95, NR2B in the hippocampus of APP/PS1 mice with control or L-DL KD (*n* = 3 per group), determined by immunoblotting (**g**) and densitometric analyses (**h**). GAPDH was included as an input control. Statistical analysis was performed using two-tailed Student’s *t*-test; * *P* < 0.05, ** *P* < 0.01, *** *P* < 0.001; error bars denote SEM

To investigate how L-DL OE leads to improved cognition in APP/PS1 mice, we next examined the alternative splicing pattern of CAMKV, and found that L-DL OE in APP/PS1 mice led to significantly decreased intron 4 retention and increased production of CAMKV protein (Fig. 8a, b, e, f). Moreover, synaptic proteins SNAP25, PSD95, NMDA receptor NR2B also showed significantly increased expression in L-DL OE APP/PS1 mouse hippocampus (Fig. 8e, f). In contrast, L-DL KD in APP/PS1 mice resulted in significantly increased intron 4 retention and consequently reduced production of CAMKV protein (Fig. 8c, d, g, h). Similarly, SNAP25, PSD95 and NR2B were also significantly decreased in the hippocampus of APP/PS1 mice with L-DL KD (Fig. 8g, h). Taken together, upregulation of L-DL improves the cognitive function of AD mice via modulating alternative splicing and subsequent synaptic protein production.

## Discussion

Alternative splicing of RNAs persists across life span, affecting more than a third of genes linked to neural function in human brains [7]. Mounting evidence has shown that significant changes in alternative splicing occur during aging progression and neurodegenerative diseases [11]. However, how these splicing changes affect cognition upon progression of aging and age-related neurodegenerative diseases remains elusive. Here we demonstrate that L-DL regulates cognition via modulating the alternative splicing patterns of cytoskeleton- and synapse-related genes. We first demonstrate that L-DL expression is substantially decreased in the hippocampus of aged and AD mouse brains. We also uncover that KD of L-DL in the hippocampus deteriorates cognition in WT and APP/PS1 mice, whereas introduction of L-DL into the hippocampus of APP/PS1 mice significantly improves the cognition of these mice.

Nuclear speckles are considered a storage site for splicing factors and therefore are functionally involved in splicing process [19]. Intriguingly, L-DL is specifically localized to nuclear speckles and L-DL KD leads to disrupted nuclear speckle structure, suggesting L-DL serves as a structural component and may participate in RNA splicing process. The pre-mRNA splicing process is executed by the spliceosome, a dynamic protein and RNA complex in eukaryotes [1]. Spliceosomes possess great plasticity in RNA substrate recognition and this process is mainly influenced by RNA binding proteins (RBPs). hnRNPs represent a large family of RBPs that are extensively involved in the regulation of RNA splicing. For instance, polypyrimidine tract binding proteins (PTBPs) and hnRNP K, which bind to U2AF65 with  similar binding motifs, regulate U2AF65-mediated recognition of U2 spliceosome at the 3′ splice sites and consequently influence RNA splicing [65, 66]. Additionally, hnRNP U directly binds to snRNAs and regulates the conversion of 15S U2 snRNP to 17S U2 snRNP to modulate U2 snRNP maturation and RNA splicing [67]. Here, we demonstrate that L-DL participates in RNA splicing process via modulating the loading of U2 snRNP-mediated spliceosome on the target RNAs. Consequently, the splicing activity is significantly decreased in L-DL deficient cells.

Regulatory role of hnRNP DL in RNA alternative splicing has been demonstrated previously in tumorigenesis [68], however, its role in the brain remains elusive. Our results demonstrate that L-DL is highly expressed in neurons and its expression is significantly reduced not only in aged mouse brains, but also in AD mouse brains. In support of these conclusions, levels of hnRNP DL are found reduced in both aged and AD human brains [69]. We also demonstrate that multiple alternative splicing changes occur in the hippocampus of L-DL KD mice. Importantly, exon skipping is the most dominant type of alternative splicing events. Moreover, among intron retention events, most of the segments showed increased inclusion (55.61%), which is consistent with splicing changes reported previously in aging brains [7]. Neuronal plasticity changes are tightly associated with RNA splicing changes during aging and age-related diseases [7, 13, 14]. In addition, previous studies have reported that synaptic plasticity, which is associated with hippocampus-dependent memory formation, is also regulated by microtubules dynamics [63]. Consistently, GO term analysis showed that genes with exon skipping in the hippocampus of L-DL KD mice are functionally related to cytoskeleton organization and synaptic functions.

Importantly, introduction of L-DL into the hippocampus of APP/PS1 mice significantly improves the cognitive function. Mechanistically, we demonstrate that L-DL regulates CAMKV expression in the hippocampus of mice by regulating *CAMKV* intron 4 alternative splicing. In support of this, previous reports have shown that CAMKV deficiency results in abnormal dendritic spine density, synaptic transmission in cultural hippocampal neurons [64]. In addition, hippocampal CAMKV knockdown mice exhibited attenuation in late-phase long-term potentiation (LTP) and memory deficits [64]. These findings support that L-DL regulates cognitive process through regulating the alternative splicing of *CAMKV* and its expression during brain aging and neurodegenerative diseases. Notably, levels of multiple synaptic proteins are down-regulated in APP/PS1 mouse brains [70]. We uncover that introduction of L-DL promotes expression of multiple synaptic genes in AD mouse brains, including CAMKV, SNAP25, PSD95 and NR2B. We therefore speculate that L-DL regulates the expression of multiple synapse- and cytoskeleton-related genes via modulating their RNA splicing process in AD brains of -.*CAMKV* Therefore, L-DL-mediated improvement in cognition in APP/PS1 mice is likely due to the restoration of multiple synaptic and cytoskeletal gene expression, beyond *CAMKV*, via changing the alternative splicing patterns of these genes.

## Conclusions

Aberrant alternative splicing changes are tightly associated with brain aging and age-related neurodegenerative diseases, however what triggers these aging associated splicing abnormalities and the regulatory mechanism behind it remain largely elusive. Here, we identify a RNA binding protein hnRNP L-DL, whose expression displays an age- and AD-dependent reduction in the brain. Notably, L-DL is preferentially localized to nuclear speckles, where L-DL regulates the alternative splicing of selected synapse- and cytoskeleton-related genes. Consequently, these gene expression are altered by L-DL. Importantly, L-DL introduction substantially improves cognitive decline in AD mice. Taken together, our study defines nuclear speckle specific protein hnRNP DL as a critical molecule linking RNA splicing and aging/AD associated cognitive decline, shedding light on a potential therapeutic approach for cognitive decline in aged and AD brains via correcting aberrant RNA splicing.

## Supplementary Information

**Additional file 1.**

## Data Availability

RNA-seq data used in this publication have been deposited in NCBI’s Gene Expression Omnibus and are accessible through GEO Series accession number GSE169281.

## Abbreviations

hnRNPs: Heterogeneous nuclear ribonucleoproteins
hnRNP DL: Heterogeneous nuclear ribonucleoprotein D-like
hnRNP A1: Heterogeneous nuclear ribonucleoprotein A1
hnRNP C: Heterogeneous nuclear ribonucleoprotein C
hnRNP U: Heterogeneous nuclear ribonucleoprotein U
hnRNP K: Heterogeneous nuclear ribonucleoprotein K
L-DL: Long isoform of hnRNP DL
S-DL: Short isoform of hnRNP DL
SF3B1: Splicing factor 3b subunit 1
SF3B2: Splicing factor 3b subunit 2
SF3B3: Splicing factor 3b subunit 3
U2 snRNP: U2 small nuclear ribonucleoprotein
U2AF65: U2 auxiliary factor 65 kDa subunit
U2AF35: U2 auxiliary factor 35 kDa subunit
5′SS: 5′ splice sites
3′SS: 3′ splice sites
AD: Alzheimer’s disease
PSD95: Postsynaptic density protein 95
PTBP1: Polypyrimidine tract binding protein 1
BACE1: Beta-secretase 1
Aβ: β-amyloid
PS1: Presenilin 1
SC35: /Splicing component 35 kDa
RBM25: RNA binding motif protein 25
NPM1: Nucleophosmin 1
PML: Promyelocytic leukemia protein
SNAP25: Synaptosome associated protein 25
NR2B: Glutamate ionotropic receptor NMDA type subunit 2B
SMN: Survival of motor neuron protein
GFAP: Glial fibrillary acidic protein
NeuN: Neuronal nuclear protein
MAPT: Microtubule associated protein Tau
PSI: Percent spliced in
DEGs: Differentially expressed genes
CADM1: Cell adhesion molecule 1
GIT1: G protein-coupled receptor kinase interacting protein 1
GIT2: G protein-coupled receptor kinase interacting protein 2
CHL1: Cell adhesion molecule L1 Like
CASK: Calcium/calmodulin-dependent serine protein kinase
CTTN: Cortactin
ADGRL1: Adhesion G protein-coupled receptor L1
SYNJ1: Synaptojanin 1
EPB41L3: Erythrocyte membrane protein band 4.1 like 3
CAMKV: Calmodulin kinase-like vesicle-associated protein
LTP: Long-term potentiation
