# Supplementary material for: Nuclear speckle specific hnRNP D-like prevents age- and AD-related cognitive decline by modulating RNA splicing
Source: Mol Neurodegener. 2021 Sep 22;16:66. doi: 10.1186/s13024-021-00485-w (PMC8456587; doi:10.1186/s13024-021-00485-w)
Supplement: Supplementary file 1 — Additional file 1. [file 13024_2021_485_MOESM1_ESM.pdf]

Supplementary Figure 1

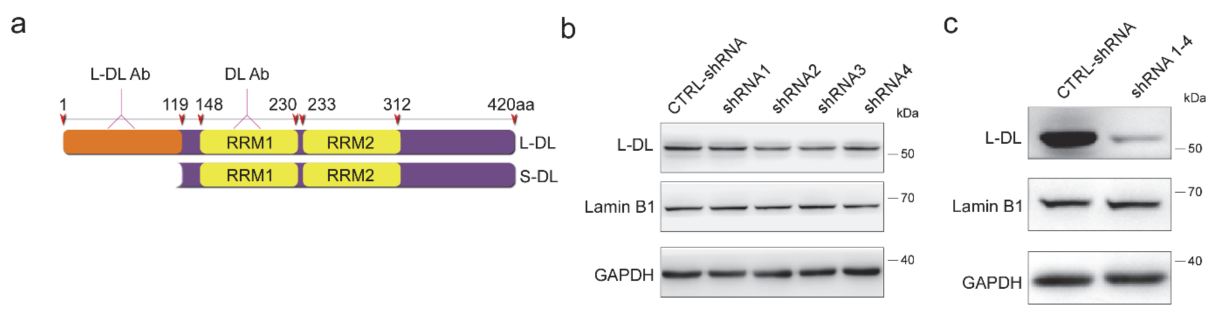

Supplementary Figure S1: (a) Diagram of long and short isoform of hnRNP DL protein (L-DL and S-DL). Epitope regions of two hnRNP DL antibodies are also shown, including a L-DL antibody that is designed to specifically recognize L-DL, and a DL antibody that is designed to recognize both L-DL and S-DL. (b) Protein levels of L-DL in N2a cells treated with control-shRNA or 4 individual shRNA targeting L-DL, namely shRNA1, shRNA2, shRNA3, and shRNA4. Lamin B1 and GAPDH were included as input controls. (c) Protein levels of L-DL in N2a cells treated with control-shRNA or 4 L-DL shRNA (shRNA1-4, shRNA1+ shRNA2+shRNA3+shRNA4). Lamin B1 and GAPDH were included as input controls.

Supplementary Figure 2

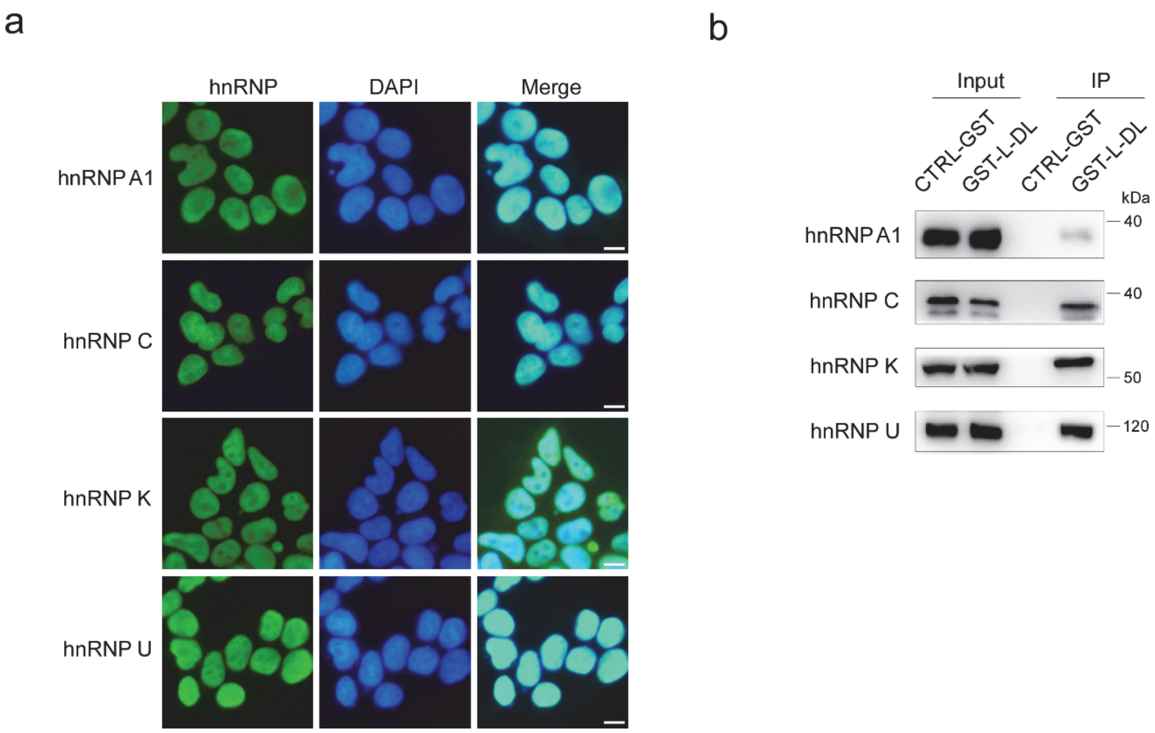

Supplementary Figure S2: (a) Representative immunofluorescence images of hnRNP A1 (Green), hnRNP C (Green), hnRNP K (Green), hnRNP U (Green) and DAPI (blue) in 293T cells. Scale bars: 10 μm. (b) CTRL-GST and GST-L-DL were introduced into 293T cells, followed by pull-down with GST agarose beads. L-DL associated proteins were subjected to immunoblotting with anti-hnRNP A1, anti-hnRNP C, anti-hnRNP K and anti-hnRNP U antibodies.

Supplementary Figure 3

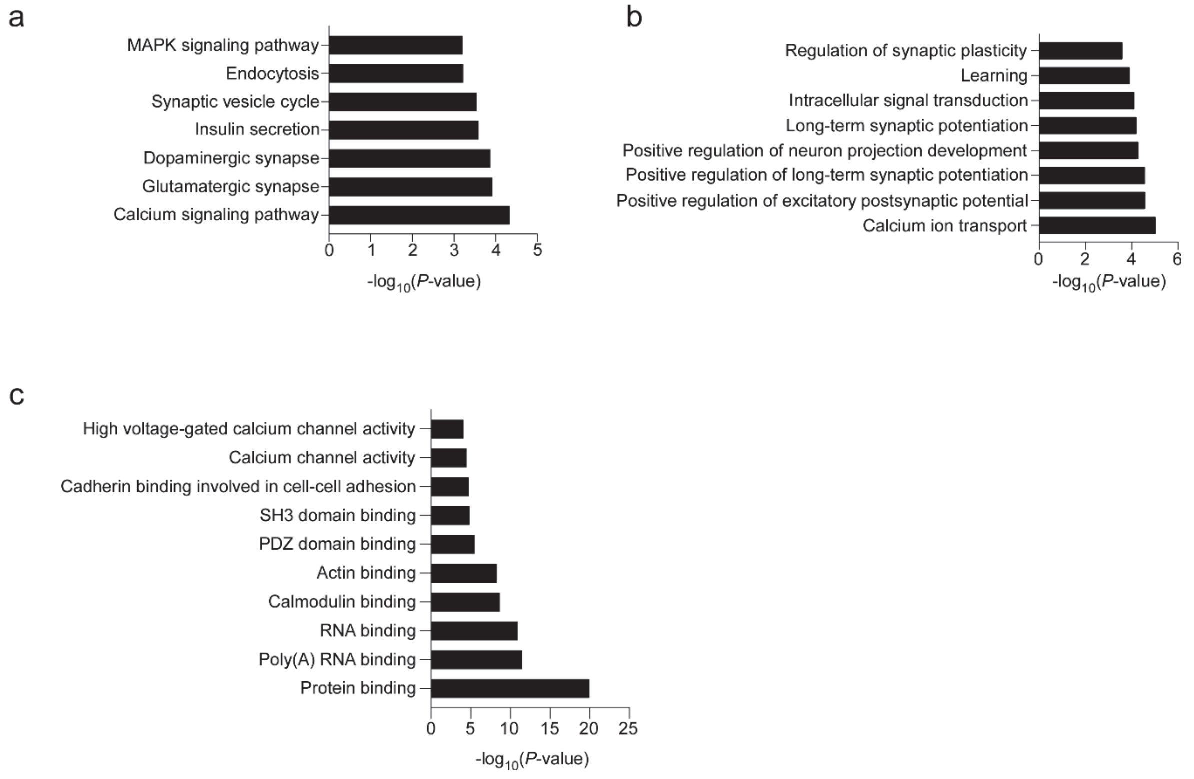

Supplementary Figure S3: (a-c) KEGG pathway analysis (a), biological process terms (b), and molecular function terms (c) for genes with exon skipping in Gene Ontology (GO) analysis (n=1,631, *P*-value < 0.001).

Supplementary Figure 4

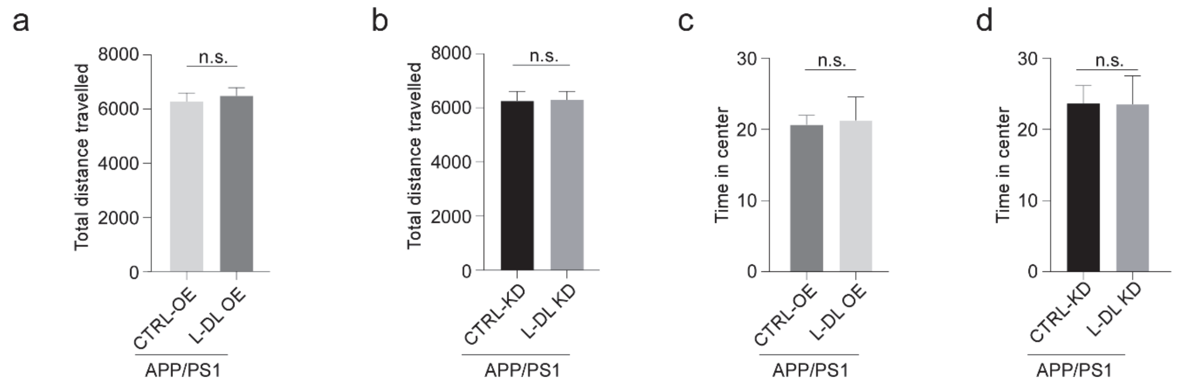

Supplementary Figure S4: In the open field task, total distance travelled (a) and time spend in the center (c) for control APP/PS1 mice and APP/PS1 mice with L-DL OE (n=15 mice per group). Total distance travelled (b) and time spend in the center (d) for control APP/PS1 mice and APP/PS1 mice with L-DL KD (n=15 mice per group). Statistical analysis was performed using two-tailed Student's *t*-test; * $P< 0.05$, ** $P< 0.01$, *** $P< 0.001$; error bars denote the SEM.

Supplementary Table S1: Primer sequences used for plasmids construction.

Plasmid	Primer sequence (5’ to 3’)	
pcDNA 3.1 (+)-GST	Forward	ATGTCCCCTATACTAGGTTA
	Reverse	GGTACACCACACTGGACTAGTGGA
pcDNA 3.1 (+)-GST-L-DL	Forward	ATGGAGGTCCCGCCCCGGCTCTC
	Reverse	TTAGTAGGGCTGGTAATTGT

Supplementary Table S2: Sequences of luciferase-WT, luciferase-Mut, tandemly arranged four L-DL shRNA for KD in the brain

**luciferase-WT**
gccaccatggaagatgccaaaaacattaagaagggcccagcgccattctacccactcgaagacgggaccgccggcgagcagctgca<br>caaagccatgaagcgctacgccctggtgcccggcaccatcgcctttaccgacgcacatatcgaggtggacattacctacgccgagtactt<br>cgagatgagcgttcggctggcagaagctatgaagcgctatgggctgaatacaaaccatcggatcgtggtgtgcagcgagaatagcttgc<br>agttcttcatgcccgtgttgggtgccctgttcatcggtgtggctgtggccccagctaacgacatctacaacgagcgcgagctgctgaacagc<br>atgggcatcagccagcccaccgtcgtattcgtgagcaagaaagggctgcaaaagatcctcaacgtgcaaaagaagctaccgatcatac<br>aaaagatcatcatcatggatagcaagaccgactaccagggcttccaaagcatgtacaccttcgtgacttcccatttgccacccggcttcaac<br>gagtacgacttcgtgcccgagagcttcgaccgggacaaaaccatcgccctgatcatgaacagtagtggcagtaccggattgcccaaggg<br>cgtagccctaccgcaccgcaccgcttgtgtccgattcagtcatgcccgcgaccccatcttcggcaaccagatcatccccgacaccgctatc<br>ctcagcgtggtgccatttcaccacggcttcggcatgttcaccacgctgggctacttgatctgcggctttcgggtcgtgctcatgtaccgcttcga<br>ggaggagctattcttgcgcagcttgcaagactataagattcaatctgccctgctggtgcccacactatttagcttcttcgctaagagcactctca<br>tcgacaagtacgacctaagcaacttgcacgagatcgccagcggcggggcgccgctcagcaaggaggtaggtgaggccgtggccaaa<br>cgcttccacctaccaggcatccgccagggctacggcctgacagaaacaaccagcgccattctgatcacccccgaaggggacgacaag<br>cctggcgcagtaggcaaggtggtgcccttcttcgaggctaaggtggtggacttggacaccggtaagacactgggtgtgaaccagcgcgg<br>cgagctgtgcgtccgtggccccatgatcatgagcggctacgttaacaaccccgaggctacaaacgctctcatcgacaaggacggctggct<br>gcacagcggcgacatcgcctactgggacgaggacgagcacttcttcatcgtggaccggctgaagagcctgatcaaatacaagggctac<br>caggtagccccagccgaactggagagcatcctgctgcaacaccccaacatcttcgacgccggggtcgccggcctgcccgacgacgatg<br>ccggcgagctgcccgccgcagtcgtcgtgctggaacacggtaaaaccatgaccgagaaggagatcgtggactatgtggccagccaggt<br>tacaaccgccaagaagctgcgcggtggtgttgtgttcgtggacgaggtgcctaaaggactgaccggcaagttggacgcccgcaagatcc<br>gcgagattctcattaaggccaagaagggcggcaagatcgccgtgaattctgcttgcaagaactggttcagtagcttaagccactttgtgatc<br>caccttaacagccacggcttccctcccgaggtggaggagcaggccgccggcaccctgcccatgagctgcgcccaggagagcggcatg<br>gatagacaccctgctgcttgcgccagcgccaggatcaacgtctaa
**luciferase-Mut**
gccaccatggaagatgccaaaaacattaagaagggcccagcgccattctacccactcgaagacgggaccgccggcgagcagctgca<br>caaagccatgaagcgctacgccctggtgcccggcaccatcgcctttaccgacgcacatatcgaggtggacattacctacgccgagtactt

cgagatgagcgttcggctggcagaagctatgaagcgctatgggctgaatacaaaccatcggatcgtggtgtgcagcgagaatagcttgc
agttcttcatgcccgtgttgggtgccctgttcatcggtgtggctgtggccccagctaacgacatctacaacgagcgcgagctgctgaacagc
atgggcatcagccagcccaccgtcgtattcgtgagcaagaaagggctgcaaaagatcctcaacgtgcaaaagaagctaccgatcatac
aaaagatcatcatcatggatagcaagaccgactaccagggcttccaaagcatgtacaccttcgtgacttcccatttgccacccggcttcaac
gagtacgacttcgtgcccgagagcttcgaccgggacaaaaccatcgccctgatcatgaacagtagtggcagtaccggattgcccaaggg
cgtagccctaccgcaccgcaccgcttgtgtccgattcagtcatgcccgcgaccccatcttcggcaaccagatcatccccgacaccgctatc
ctcagcgtggtgccatttcaccacggcttcggcatgttcaccacgctgggctacttgatctgcggctttcgggtcgtgctcatgtaccgcttcga
ggaggagctattcttgcgcagcttgcaagactataagattcaatctgccctgctggtgcccacactatttagcttcttcgctaagagcactctca
tcgacaagtacgacctaagcaacttgcacgagatcgccagcggcggggcgccgctcagcaaggaggtaggtgaggccgtggccaaa
cgcttccacctaccaggcatccgccagggctacggcctgacagaaacaaccagcgccattctgatcacccccgaaggggacgacaag
cctggcgcagtaggcaaggtggtgcccttcttcgaggctaaggtggtggacttggacaccggtaagacactgggtgtgaaccagcgcgg
cgagctgtgcgtccgtggccccatgatcatgagcggctacgttaacaaccccgaggctacaaacgctctcatcgacaaggacggctggct
gcacagcggcgacatcgcctactgggacgaggacgagcacttcttcatcgtggaccggctgaagagcctgatcaaatacaagggctac
caggtaagtatcaaggttacaagacaggtttaaggagaccaatagaaactgggcttgtcgagacagagaagactcttgcgtttctgatagg
cacctattggtcttactgacatccactttgcctttctctccacaggtagccccagccgaactggagagcatcctgctgcaacaccccaacatct
tcgacgccggggtcgccggcctgcccgacgacgatgccggcgagctgcccgccgcagtcgtcgtgctggaacacggtaaaaccatga
ccgagaaggagatcgtggactatgtggccagccaggttacaaccgccaagaagctgcgcggtggtgttgtgttcgtggacgaggtgccta
aaggactgaccggcaagttggacgcccgcaagatccgcgagattctcattaaggccaagaagggcggcaagatcgccgtgaattctgc
ttgcaagaactggttcagtagcttaagccactttgtgatccaccttaacagccacggcttccctcccgaggtggaggagcaggccgccggc
accctgcccatgagctgcgcccaggagagcggcatggatagacaccctgctgcttgcgccagcgccaggatcaacgtctaa

tandemly arranged and H1 promoter driven four L-DL shRNA for L-DL KD in the brain.

gaacgctgacgtcatcaacccgctccaaggaatcgcgggcccagtgtcactaggcgggaacacccagcgcgcgtgcgccctggcagg
aagatggctgtgagggacaggggagtggcgccctgcaatatttgcatgtcgctatgtgttctgggaaatcaccataaacgtgaaatgtctttg
gatttgggaatcttataagttctgtatgagaccacttgcatccAGCAAGGGACTGAGCCGAACCTTCCTGTCAGAGTT
CGGCTCAGTCCCTTGCTTTTTTgaacgctgacgtcatcaacccgctccaaggaatcgcgggcccagtgtcactaggcggg
aacacccagcgcgcgtgcgccctggcaggaagatggctgtgagggacaggggagtggcgccctgcaatatttgcatgtcgctatgtgttc
tgggaaatcaccataaacgtgaaatgtctttggatttgggaatcttataagttctgtatgagaccacttgcatccCCAACGGCTCTCA
AGGCAAGCTTCCTGTCAGACTTGCCTTGAGAGCCGTTGGTTTTTgaacgctgacgtcatcaacccgctcca
aggaatcgcgggcccagtgtcactaggcgggaacacccagcgcgcgtgcgccctggcaggaagatggctgtgagggacaggggagt

ggcgccctgcaatatttgcatgtcgctatgtgttctgggaaatcaccataaacgtgaaatgtctttggatttgggaatcttataagttctgtatgag
accacttgcatccCCTCATCCGGAGCGCGGCGACTTCCTGTCAGATCGCCGCGCTCCGGATGAGGT
TTTTgaacgctgacgtcatcaacccgctccaaggaatcgcgggcccagtgtcactaggcgggaacacccagcgcgcgtgcgccctg
gcaggaagatggctgtgagggacaggggagtggcgccctgcaatatttgcatgtcgctatgtgttctgggaaatcaccataaacgtgaaat
gtctttggatttgggaatcttataagttctgtatgagaccacttgcatccGAACAATGGCGGCGGCACATCTTCCTGTCAG
AATGTGCCGCCGCCATTGTTCTTTTT

Supplementary Table S3: sgRNA sequences targeting human or mouse L-DL

Human sgRNA sequence (5' to 3')	
H-sgRNA1	AAGGAGAGAGGCCACGCGTG
H-sgRNA2	GGCGGCACATGGGAAAGCCT
H-sgRNA3	GGAGAGAGGCCACGCGTGAG
H-sgRNA4	GCTAAAGTAGCGGGAGCGGA
Mouse sgRNA sequence (5' to 3')	
M-sgRNA1	AGCAAGGGACTGAGCCGAAC
M-sgRNA2	CCAACGGCTCTCAAGGCAAG
M-sgRNA3	CCTCATCCGGAGCGCGGCGA
M-sgRNA4	GAACAATGGCGGCGGCACAT

Supplementary Table S4: L-DL associated proteins identified in MS

Accession	Gene names	Sequence coverage (%)	# Unique Peptides	# PSMs
P61978	hnRNP K	45.14	20	48
Q08211	DHX9	25.35	33	39
P22626	hnRNP A2B1	40.51	12	32
P09651	hnRNP A1	34.14	9	22
P14866	hnRNP L	34.3	15	19
Q00839	hnRNP U	13.45	11	14
Q9NZI8	IGF2BP1	22.18	11	14
Q96I24	FUBP3	16.78	9	14
P51991	hnRNP A3	16.67	3	12
Q13151	hnRNP A0	14.1	3	10
Q4VCS5	AMOT	9.32	9	9
Q07666	KHDRBS1	11.96	7	9
O14979	hnRNP DL	12.62	5	9
P31942	hnRNP H3	20.81	8	9
Q96KR1	ZFR	8.66	7	8
Q9Y5B9	SUPT16H	6.11	7	7
O43390	hnRNP R	12.01	3	7
Q9NZB2	FAM120A	7.16	6	6

P17844	DDX5	8.96	6	6
O60506	SYNCRIP	11.08	2	6
Q15717	ELAVL1	16.56	5	6
Q15393	SF3B3	3.62	5	5
P35637	FUS	7.03	4	5
P43243	MATR3	4.25	3	4
P02768	ALB	6.08	3	4
P08621	SNRNP70	9.61	4	4
P16152	CBR1	16.61	4	4
P07910	hnRNP C	9.48	4	4
Q9UKM9	RALY	11.76	4	4
Q8N163	CCAR2	3.36	3	3
O60814	HIST1H2BK	11.9	1	3
Q15424	SAFB	2.73	3	3
Q7L2E3	DHX30	2.43	3	3
O60264	SMARCA5	2.47	3	3
P26368	U2AF65	5.47	3	3
Q9UHX1	PUF60	6.08	3	3
P22087	FBL	9.35	3	3
P06748	NPM1	7.82	3	3

Q14978	NOLC1	2.86	2	2
Q9BXP5	SRRT	2.28	2	2
P09874	PARP1	2.17	2	2
P78347	GTF2I	1.9	2	2
P81605	DCD	20	2	2
Q13435	SF3B2	2.01	2	2
Q9UQE7	SMC3	1.64	2	2
Q8WXF1	PSPC1	3.63	2	2
O76021	RSL1D1	3.47	2	2
Q14739	LBR	3.09	2	2
P16403	HIST1H1C	9.86	2	2
P29692	EEF1D	7.47	2	2
Q99623	PHB2	5.35	2	2
Q01081	U2AF35	6.67	2	2
P68104	EEF1A1	8.66	2	2
Q6Y7W6	GIGYF2	0.69	1	1
P62937	PPIA	10.91	1	1
Q92900	UPF1	0.89	1	1
Q9H0A0	NAT10	0.78	1	1
P0CG48	UBC	11.82	1	1

O75533	SF3B1	0.84	1	1
P31151	S100A7	10.89	1	1
P49756	RBM25	1.3	1	1
P60709	ACTB	6.13	1	1
Q9Y2W1	THRAP3	0.73	1	1
P10809	HSPD1	4.36	1	1
Q9H307	PNN	1.53	1	1
Q8IY81	FTSJ3	0.94	1	1
O15042	U2SURP	0.78	1	1
Q8IXT5	RBM12B	0.9	1	1
P22492	HIST1H1T	5.31	1	1
P25311	AZGP1	3.36	1	1
P02545	LMNA	1.36	1	1
P52272	hnRNP M	1.51	1	1
Q6NXT2	H3F3C	5.19	1	1
Q12906	ILF3	1.45	1	1
P12273	PIP	5.48	1	1
Q13283	G3BP1	2.36	1	1
O95969	SCGB1D2	10	1	1
P08670	VIM	2.15	1	1

Q9HD64	XAGE1A	7.41	1	1
Q9BY76	ANGPTL4	1.48	1	1
Q9P2B4	CTTNBP2NL	0.94	1	1
Q12800	TFCP2	2.39	1	1
Q9H853	TUBA4B	5.81	1	1
Q07021	C1QBP	4.61	1	1
Q15366	PCBP2	3.84	1	1
Q9P035	HACD3	3.87	1	1
Q16629	SRSF7	5.04	1	1
Q07955	SRSF1	4.03	1	1

Supplementary Table S5: Primers used for alternative splicing analysis using PCR-based approach

Gene	Prime sequence (5’ to 3’)	
GIT2	Forward	CAAGCCACTCGTCCCTAAA
	Reverse	CACAGGCAGTGTTGTCATAATC
CTTN	Forward	CTTTGGAGGAAAGTTTGGTGTG
	Reverse	CTTGTCCATCCGATCCTTCTG
PSD95	Forward	CAGTACAACAGCCACCTCTAC
	Reverse	AACTTGGCAAGCAAACCTTC
MAPT	Forward	GAAGCAGGCATCGGAGAC
	Reverse	CGTCATTTCCTGTCCTGTCT
GIT1	Forward	GCTAGTTGAGTGCCAGTATGAG
	Reverse	GTCACCAGGGTGCTATGATTT
EPB41L3	Forward	CCACAACAAAGGGCATCTCT
	Reverse	TCAATCATGGGTGCATCCTC
AGRIN	Forward	GCAGTAGATGTAGCGCCTTT
	Reverse	GGTCATAGCTCAGTTGTAGG
CASK	Forward	GGGAAATGCGAGGGAGTATT
	Reverse	GCTGCTCTTGTTTGGTCTTC
CHL1	Forward	GCATCTCTCCAACCTCAACT

	Reverse	CTTTCCACCTCTGTTCCTCT
CADM1	Forward	GGGTGAGAGTCGATGATGAA
	Reverse	GAGTTGTTCTGTCCTCCTTC
SYNJ1	Forward	CCAGAAGGATTCTTCCCAG
	Reverse	GTCTAGCCGCACCGTATC
ADGRL1	Forward	GTGCCATTGCACTGCTCTT
	Reverse	GGTGCTGTTGATGTCACCT
CAMKV	Forward	TGCAGCTGGTAGATGTGTTT
	Reverse	GAGGTTCCTGTGCACAATCT
